# Intergrative Taxonomic Study of the *Frullania parvistipula* Complex with a Modern Circumscription of the Section *Trachycolea* (Frullaniaceae, Marchantiphyta)

**DOI:** 10.3390/plants13172397

**Published:** 2024-08-27

**Authors:** Yuriy S. Mamontov, Anna A. Vilnet, John J. Atwood, Nadezhda A. Konstantinova

**Affiliations:** 1 Tsitsin Main Botanical Garden, Russian Academy of Sciences, Botanicheskaya 4, Moscow 127276, Russia; 2 Polar-Alpine Botanical Garden-Institute, Russian Academy of Sciences, Kirovsk 184256, Russia; anya_v@list.ru (A.A.V.); nadya50@list.ru (N.A.K.); 3 Missouri Botanical Garden, 4344 Shaw Boulevard, St. Louis, MO 63110, USA; john.atwood@mobot.org

**Keywords:** Holarctic, hybridization, liverworts, molecular phylogeny, morphology, ITS1-2 nrDNA, *trn*L-F cpDNA

## Abstract

*Frullania* (subg. *Trachycolea*) sect. *Trachycolea* has been studied using integrative taxonomy methods and utilizing sampling from almost all areas of distribution of the species previously referred to this section. A phylogenetic analysis based on nuclear ribosomal ITS1-2 and chloroplast *trn*L-F sequence data and a morphological study reveal a wide range of morphological variability within specimens that has largely disguised the overall taxonomic diversity. *Frullania parvistipula*, previously regarded as a widespread species, has been found to represent a group of separate species within different sections of *F.* subg. *Trachycolea*: *F. caucasica* and *F. conistipula* in *F.* sect. *Trachycolea*, *F. parvistipula* in *F.* sect. *Australes*, and *F. fukuzawana* in *F*. sect. *Integristipulae* II. Illustrations of the type specimens of *F. conistipula*, *F. fukuzawana*, and *F. parvistipula*, as well as illustrations of the sequenced specimens belonging to two of the discussed species (*F. conistipula* and *F. parvistipula*), are provided. The morphological differences separating the highly similar *F. caucasica*, *F. conistipula*, *F. fukuzawana*, *F. koponenii*, and *F. parvistipula* are discussed. A dichotomous key is presented for accepted species. New combinations are provided for two taxa.

## 1. Introduction

A growing number of integrative taxonomic studies on bryophytes, especially leafy liverworts, have shown that the sole reliance on morphological data in systematics may result in an underestimate of species diversity [1,2,3,4], as well as insufficiently resolved infrageneric [5,6] and intrafamiliar relationships [7,8,9], due to the subtleness and convergence of morphological characteristics. As seen in recent integrative studies on the hyperspecious genus *Frullania* Raddi, the coupling of morphological and molecular phylogenetic data can provide new insights into taxonomic relationships and species delimitations. Hentschel et al. [10] found *F. orientalis* Sande Lac. (=*F.* subg. *Orientales* (Verd.) S.Hatt.), a species of intermediate morphology, to be nested within *F.* subg. *Trachycolea* Spruce in a sister relationship with *F. ferdinandi-muelleri* Steph., whose morphology, at least superficially, seems dissimilar. Similarly, *F.* (subg. *Trachycolea*) subsect. *Inflatae* R.M.Schust. was found by Mamontov et al. [11] to be polyphyletic and composed of three lineages, with species of the first lineage forming their own subgenus (*F.* subg. *Frullaniopsis* J.J. Atwood, Vilnet & Mamontov), whereas species of the other two lineages belonging to the subgenera *F.* subg. *Frullania* and *F.* subg. *Chonanthelia* Spruce. Lastly, an integrative study on *F. diversitexta* Steph., the sole representative of the monospecific *F.* subg. *Diversitextae* (Kamim.) S.Hatt., found that its phylogenetic relationship resolved within *F.* subg. *Trachycolea*, as sister to *F. plana* Sull., with which it shares several morphological peculiarities [12].

Here, we consider the taxonomy of *F*. (subg. *Trachycolea*) sect. *Trachycolea* (Spruce) Grolle and the *F. parvistipula* Steph. complex, which was earlier considered to be included within this section. The *F. parvistipula* complex consists of *F. parvistipula* and taxa that were synonymized with it or with *F. muscicola* Steph., and are similar in having caducous leaves, a *dilatata*-type leaf, and underleaf morphology [11] (p. 210), as well as dioicous sexuality. The taxonomy of *F*. sect. *Trachycolea* and the *F. parvistipula* complex has been controversial due to its both broadly and narrowly defined morphological circumscriptions.

Twenty species are noted in Söderström et al. [13] within *F*. sect. *Trachycolea* (as *F*. sect. *Frullania*), of which *F. dilatata* (L.) Dumort. is the type species. Bombosch et al. [14] found *F. appalachiana* R.M.Schust., *F. azorica* Sim-Sim, Sergio, Mues & Kraut, *F. eboracensis* Lehm., *F. parvistipula*, and *F. virginica* Lehm. to be closely related to *F. dilatata* based on a molecular phylogenetic study of nuclear and chloroplast DNA markers. The core of the section was therefore determined to include these six species, all of which share a *dilatata*-type morphology and dioicous sexuality. The majority of the other 14 species attributed to *F*. sect. *Trachycolea* were sequenced in Hentschel et al. [10], Bombosch et al. [14], and Mamontov et al. [11], but almost all were found to be phylogenetically distant from *F. dilatata*. By comparison, most of these species have monoicous (autoicous or paroicous) sexuality and/or morphology that is divergent from the *dilatata*-type. The autoicous *F. catalinae* A.Evans, *F. oakesiana* Austin, *F. stylifera* (R.M.Schust.) R.M.Schust., and *F. takayuensis* Steph., as well as the dioicous *F. bolanderi* Austin, all lack intermediate thickenings in their leaf lobe cells and phylogenetically were attributed to a separate subgenus, *F.* subg. *Frullaniopsis* [11]. Furthermore, Mamontov et al. [11] considered the autoicous (sometimes paroicous) *F. sabaliana* R.M.Schust. to belong to *F*. subg. *Chonanthelia*. On the other hand, the dioicous *F. ericoides* (Nees) Mont. has been found to be a paraphyletic taxon, with the sequenced specimens showing a close relationship with *F*. sect. *Acutilobae* Verd. [10] (p. 150). In *F. ericoides* s.l., the underleaves are divergent from the *dilatata*-type morphology, in being (2–)2.5–3.5(–5)× the stem width [15] (p. 229). All lineages of *F. ericoides* s.l. that were sequenced in Hentschel et al. [10] are therefore not considered here to belong to *F*. sect. *Trachycolea*. Finally, the ocelli-bearing *F. fragilifolia* (Taylor) Gottsche and the autoicous *F. chilcootiensis* Steph. (as *F. hattoriana* J.D.Godfrey & G.Godfrey) have since been placed into *F.* subg. *Frullania* [10,11,16]. In both of these species, the leaf lobules are divergent from the *dilatata*-type, being more narrowly galeate in outline [17] (p. 472), [16] (p. 3).

The phylogenetic relationships of the remaining seven taxa attributed to *F.* sect. *Trachycolea* by Söderström et al. [13], namely the Holarctic *F. dilatata* subsp. *asiatica* S.Hatt., *F. koponenii* S.Hatt., *F. muscicola* (including the previously synonymized *F. aeolotis* var. *aberrans* C.Massal.), and *F. subdilatata* C.Massal.; the Neotropical *F. semivillosa* Lindenb. & Gottsche; the Antarctic *F. fuegiana* Steph.; and the recently described *F. tibetica* Mamontov & J. J. Atwood [18], remain unresolved. Since some of these taxa (including *F. muscicola*) have morphology that clearly diverges from the *dilatata*-type, a better understanding of the morphological range of *F*. sect. *Trachycolea* must be determined.

Whereas the studies of Kamimura [19] and Schuster [15] have favored informal (*F. muscicola* expression *parvistipula*) and formal infraspecific ranks (*F. eboracensis* subsp. *eboracensis*, *F. eboracensis* subsp. *virginica* (Lehm.) R. M. Schust., and *F. eboracensis* subsp. *parvistipula* (Steph.) R. M. Schust.) of *F. parvistipula*, narrower species concepts (e.g., the treatment of *F. eboracensis*, *F. parvistipula*, and *F. virginica* as separate species) have since been advocated for by Bombosch et al. [14]. Among the species studied by Bombosch et al. [14] are specimens named *F. parvistipula* from Europe and North America, although there is some ambiguity regarding the name of the *F. parvistipula* clade in their study, since the sampling did not include specimens from Japan, where *F. parvistipula* was first described, or other East Asian regions, where *F. parvistipula* is known to be distributed. The taxonomic relationship between *F. muscicola* and *F. parvistipula* is also open to inquiry since the former species was never included in a molecular study.

In order to better clarify the taxonomy of *F*. sect. *Trachycolea* and the *F. parvistipula* complex, it is necessary to delimit the morphological features that distinguish species within these groups and determine the status and sectional affiliation of the taxa previously attributed to *F*. sect. *Trachycolea* by Söderström et al. [13]. Specimens determined as *F. aeolotis* var. *aberrans*, *F. caucasica*, *F. dilatata*, *F. fukuzawana*, *F. koponenii*, *F. muscicola*, and *F. parvistipula* from the U.S.A., Europe, the Caucasus, Siberia, the Russian Far East, and China were studied using an integrative approach. The combined dataset utilizes the sequences of *F. appalachiana*, *F. azorica*, *F. dilatata*, *F. eboracensis*, *F. virginica*, and *F. parvistipula* generated by Bombosch et al. [14] and therefore contains almost all species of *F.* sect. *Trachycolea* as circumscribed by Söderström et al. [13] (except for the species belonging to *F*. subg. *Chonanthelia*, *F*. subg. *Frullania*, and *F*. subg. *Frullaniopsis* in Mamontov et al. [11]). Indeed, a larger number of distinct taxa within *F.* subg. *Trachycolea* was found. Particularly, four taxa require resurrecting from synonymy: *F. aeolotis* var. *aberrans*, *F. caucasica*, *F. conistipula* Steph., and the invalid *F. fukuzawana* Steph. ex Bonner. The latter’s name is validated here as *F. fukuzawana* Steph. ex Mamontov, J. J. Atwood & Vilnet sp. nov., while *F. aeolotis* var. *aberrans* and *F. dilatata* subsp. *asiatica* are recognized as the distinct species *F. aberrans* (C. Massal.) Mamontov, Vilnet & J. J. Atwood comb. et stat. nov. and *F. asiatica* (S. Hatt.) Mamontov & J. J. Atwood comb. et stat. nov.

## 2. Materials and Methods

### 2.1. Morphological Study

The specimens used for this study were examined using light microscopes equipped with digital cameras. In order to better illustrate the three-dimensional objects, photomicrographs were combined from several optical sections using the stacking software HeliconFocus 8.2.0 (https://www.heliconsoft.com/software-downloads/ accessed on 9 September 2023) [20] and then reconstructed in line drawings. The type specimens of *F. aeolotis* var. *aberrans* (G!, LE!, NICH!), *F. caucasica* Steph. (LE!), *F. conistipula* (G!), *F. dilatata* subsp. *asiatica* (NICH!), *F. fukuzawana* (original material, G!), *F. koponenii* (NICH!), *F. muscicola* (G!, NICH!), *F. parvistipula* (G!), and *F. subdilatata* (G!) were studied as references to the morphological circumscriptions associated with these taxa. The taxonomic circumscriptions presented here have been derived from the overall studied suite of specimens, including those used in the molecular analysis, as well as type specimens and specimens cited in the literature.

### 2.2. Taxa Sampling

ITS1-2 nrDNA and *trn*L-F cpDNA sequence data for 66 specimens have been newly obtained and analyzed together with GenBank accessions for 35 specimens previously published in Hentschel et al. [10], Bombosch et al. [14], and Atwood et al. [12]. The ingroup of the combined dataset includes representatives of sections *Trachycolea*, *Australes* Verd., *Integristipulae* II., *Acutilobae* Verd., and *Planae* R. M. Schust. within *F*. subg. *Trachycolea*, as well as several other species which are still unclassified within the current sectional subdivisions of the subgenus. The species *F. moniliata* (Reinw., Blume & Nees) Mont. with *F*. subg. *Frullania* was chosen as the outgroup taxon. The voucher details and GenBank accession numbers for all tested specimens are listed in Table 1.

### 2.3. DNA Isolation, Amplification, and Sequencing

DNA was extracted from dried liverwort tissue using the DNeasy Plant Mini Kit (QIAGEN, Hilden, Germany). The ITS1-2 and *trn*L-F loci were amplified and sequenced with pairs of primers, as described in White et al. [21] and Taberlet et al. [22]. PCR was carried out in 20 µL volumes with the following amplification cycles: 3 min at 94 °C, 30 cycles (30 s 94 °C, 40 s 56 °C, 60 s 72 °C) and 2 min of final extension time at 72 °C. The amplified fragments were visualized on 1% agarose TAE gels by EthBr staining, purified using the Cleanup Mini Kit (Evrogen, Moscow, Russia), and used as a template in sequencing reactions with the ABI Prism BigDye Terminator Cycle Sequencing Ready Reaction Kit (Applied Biosystems, Waltham, MA, USA) following the standard protocol provided for the 3730 DNA Analyzer (Applied Biosystems, Waltham, MA, USA).

### 2.4. Phylogenetic Analysis

The newly obtained ITS1-2 and *trn*L-F nucleotide sequences were assembled and then automatically aligned with the downloaded accessions from GenBank in BioEdit 7.0.1 [23] using the option of full multiple alignment with default settings for gaps and extension weights in the ClustalW tool. The preliminary phylogenetic estimation of ITS1-2 and *trn*L-F datasets revealed incongruences due to the positions of five specimens from the Russian part of the Caucasus (four from the Krasnodar Territory and one from the Chechen Republic), which were excluded from subsequent estimations of the combined dataset ITS1-2+*trn*L-F. The combined dataset ITS1-2+*trn*L-F was manually corrected, all positions of the final alignment were included in the phylogenetic estimations, and absent data at the end of the regions and unsequenced loci were coded as missing.

The phylogeny was reconstructed with three analytical procedures: the maximum parsimony method (MP) with the TNT v. 1.5 program [24], the maximum likelihood method (ML) with PhyML 3.0 [25], and the Bayesian approach (BA) with MrBayes v. 3.2.1 [26].

In the MP analysis provided with New Technology Search, the minimum-length tree was searched for in five reiterations, the resampling procedure was performed with 1000 bootstrap replicates, gaps were treated as missing, and the default settings were used for other parameters. The best-fit evolutionary model of nucleotide substitutions for ML analysis—TN+I+G—was determined in ModelGenerator [27]. In addition, in the ML analysis, the rate heterogeneity among sites was modelled using a gamma distribution with four rate categories, and a bootstrap procedure was implemented with 500 replicates. The stopping frequency criterion for bootstrapping [28] for our dataset suggested that 250 replicates were enough for reaching BS convergence with Pearson average ρ100 = 0.993673 realized in RAxML v.7.2.6 [29]. For the Bayesian analysis, the ITS1-2 and *trn*L-F partitions of the alignments were separately assigned the most common GTR+I+G model as recommended by the program authors, and the gamma distributions were approximated using four categories. Two independent runs of the Metropolis-coupled ΜCMC with four chains were used, and the two starting trees were chosen randomly. The number of generations was 10 million, and trees were saved every 1000th generation. The average standard deviation of split frequencies between the two runs was 0.003211 at the end of the run. The software tool Tracer v. 17 [30] revealed the effective sample size (ESS) to be 11,984.4027 and the auto-correlation time (ACT) to be 1501.9689 for our data. The first 1000 trees as determined by Tracer were discarded in each run, and 18,000 trees from both runs were sampled after burning. Bayesian posterior probabilities were calculated from trees sampled after burn-in.

The average pairwise *p*-distances calculated in Mega 11 [31] were obtained to estimate the level of infraspecific and infrageneric variability of ITS1-2 and *trn*L-F for the species of *F*. sect. *Trachycolea* (Table 2).

## 3. Results

**Figure 1:**
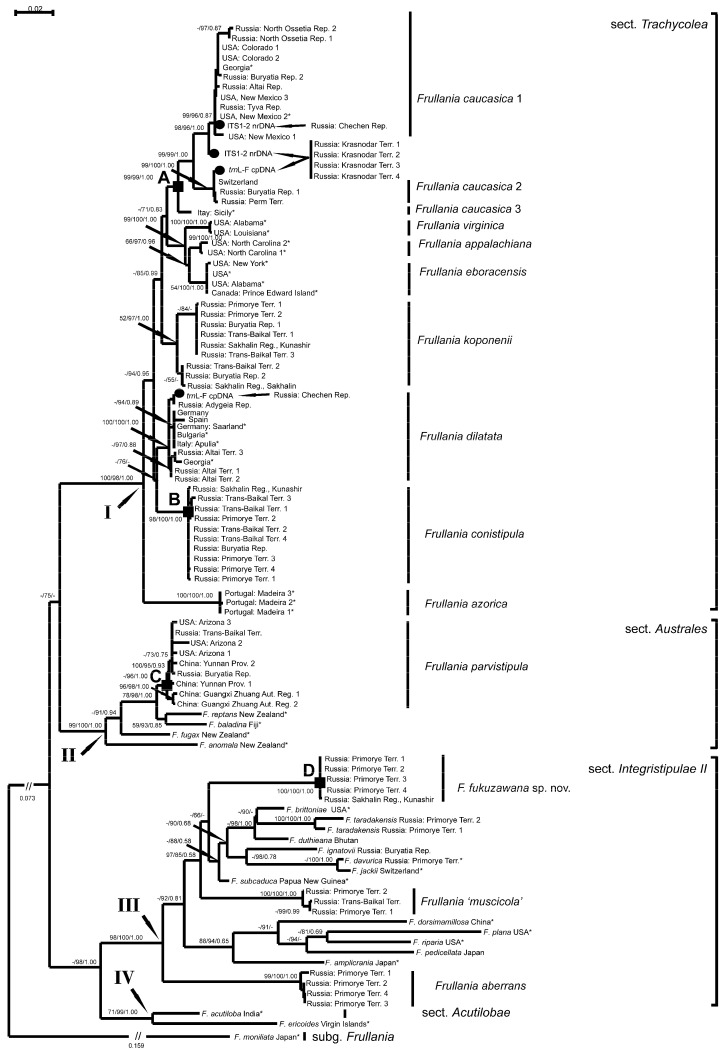
Phylogram obtained using the maximum likelihood approach for the *F*. sect. *Trachycolea* and related taxa. Asterisks mark specimens, the sequences of which were downloaded from GenBank. The placement of specimens with hybrid origin is marked by dots, and notes are provided.

For the 65 sampled *Frullania* specimens, 63 sequences of ITS1-2 and 56 sequences of *trn*L-F were generated (Table 1). In total, the newly generated alignment contained nucleotide sequence data from 96 specimens and consists of 1808 sites, of which 1235 belong to ITS1-2, and 573 belong to *trn*L-F. The number of conservative positions in ITS1-2 and *trn*L-F is 617 (49.95%) and 467 (81.50%), respectively, the number of variable positions is 559 (45.26%) and 106 (18.50%), and the number of parsimony-informative positions is 404 (33.11%) and 58 (10.12%). The MP analysis yielded 21 equally parsimonious trees with a length of 2806 steps, with CI = 0.670704 and RI = 0.888261 calculated in Mega 5.1. The ML calculation resulted in a single tree; the arithmetic means of Log likelihood was −9809.423. The arithmetic means of Log likelihoods in the BA analysis for both runs sampled were −9649.90 and −9650.67, respectively. All of the obtained topologies achieved by the three methods are highly congruent. The ML tree for 100 specimens, with indicated bootstrap support values from MP/ML calculations and values of Bayesian posterior probabilities more than 50% (0.50), is shown in Figure 1. The positions of the five specimens excluded from the analyses of the combined dataset (see Section 2.4. *Phylogenetic Analysis*) are marked (black circles) according to the results of the separate ITS1-2 and *trn*L-F estimations.

### 3.1. Main Clades

The presented phylogeny of *F*. subg. *Trachycolea* reveals four main clades, labeled with Roman numerals (Figure 1). The first clade (I) corresponds to *F*. sect. *Trachycolea* (BS in MP = 100%, BS in ML = 98%, PP in BA = 1.00, abbreviated as 100/98/1.00) since it includes *F. dilatata* and other closely related species. The obtained topology for *F*. sect. *Trachycolea* resembles those published and discussed by Hentschel et al. [10] and Bombosch et al. [14]. The second clade (II) contains representatives of *F*. sect. *Australes* Verd. (99/100/1.00). The third clade (III) includes species of *F*. sect. *Integristipulae* II sensu Hentschel et al. [10], namely *F. davurica* Hampe ex Gottsche, Lindenb. & Nees and *F. jackii* Gottsche. This clade also contains several other species sequenced in Hentschel et al. [10], namely *F. amplicrania* Steph., *F. brittoniae* A.Evans, *F. dorsimamillosa* Mamontov, Hentschel, Sofronova & Potemkin (as *F. fuscovirens* Steph. var. *gemmipara* (R.M.Schust. & S.Hatt.) S.Hatt. & P.J.Lin in Hentschel et al. [10]), *F. duthieana* Steph. (as *F. duthiana* in Hentschel et al. [10]), *F. riparia* Hampe ex Lehm., *F. subcaduca* S.Hatt., and *F. taradakensis* Steph. These species are closely related to *F. davurica* and *F. jackii* but were not attributed to a section in Hentschel et al. [10]. In general, clade III mainly corresponds to the clade composed of the same species in Henschel et al. [10], where that clade was also supported. The phylogeny obtained by Hentschel et al. [10] and the one presented here, therefore, suggest affiliation of the species closely related to *F. davurica* and *F. jackii* to the same section. This section requires a morphological circumscription and the selection of a formal name. However, until this taxonomic issue is resolved, clade III is named hereafter as *F*. sect. *Integristipulae* II. An interesting issue is that *F. plana* Sull., the representative of monospecific *F*. sect. *Planae* R.M.Schust., is nested in the presented phylogeny within the clade of *F*. sect. *Integristipulae* II, whereas in the phylogeny obtained by Hentschel et al. [10], this species was found in a sister relationship with this group but was not nested within it. To resolve this issue, further studies on the taxonomy of *F*. subg. *Trachycolea* based on an expanded sampling of taxa, including species of *F*. sect. *Ornitocephalae* II sensu Hentschel et al. [10] and *F*. sect. *Diversitextae* (Kamim.) J.J.Atwood, Vilnet & Mamontov, are needed.

The fourth main clade (IV) (77/99/1.00) includes *F. acutiloba* Mitt., the type species of *F*. sect. *Acutilobae* Verd., and a haplotype of *F. ericoides* (Nees) Mont. The latter taxon was shown in Hentschel et al. [10] to be paraphyletic but has not since been classified there. The grouping of *F. acutiloba* and several unrelated haplotypes named *F. ericoides* into one clade is supported in Hentschel et al. [10], where that major clade contains species belonging to *F*. sect. *Integristipulae* I and *F*. sect. *Ornitocephalae* I sensu Hentschel et al. [10], as well as *F*. sect. *Irregulares* Verd. and *F*. sect. *Orientales* Verd. The sister phylogenetic relationships of all of these taxa, and their morphological similarity in having more or less rostrate leaf lobules, suggest their uniting into a single section. However, this taxonomic issue requires further study to circumscribe this group and determine priority among the previously published sectional names associated with species in this group.

### 3.2. Subclades of Taxa of the Frullania parvistipula Complex 

The specimens of the *F. parvistipula* complex consist of four subclades (marked with black squares and labeled A, B, C, and D). Species such as *F. appalachiana*, *F. eboracensis*, and *F. koponenii*, although morphologically similar, are not considered to belong to the *F. parvistipula* complex because they have never been synonymized with *F. parvistipula* or *F. muscicola*. 

The specimens from Russia, China, and the U.S.A. are morphologically most similar to the type of *F. parvistipula* representing subclade C within clade II (*F*. sect. *Australes*). Despite the wide distributon of *F. parvistipula*, the level of its infraspecific sequence variability (0.8/0.3) is quite low compared to the variability within the also widely distributed *F. caucasica* s.l. (see below).

The terminal part of clade I, the subclade A, contains several specimens from the U.S.A., Italy, and Georgia, which were previously included in phylogenies by Henstchel et al. [10] and Bombosch et al. [14] as *F. eboracensis* subsp. *parvistipula* and *F. parvistipula.* This subclade also includes several newly sequenced specimens from Switzerland and Russia, and splits into three closely related groups of haplotypes, the specimens of which (including the ones from the Caucasus) are morphologically most similar to the type specimen of *F. caucasica*. Subclade A is therefore treated as *F. caucasica*. Hattori [32] considered *F. caucasica* to be synonymous with *F. parvistipula*, and Schuster [15] regarded it to be a subspecies of *F. eboracensis*. The three taxa are in fact morphologically and phylogenetically distinct, which excludes the synonymy of *F. caucasica* with *F. parvistipula* and its recognition at the subspecific rank. We assign the group containing the Caucasus specimens to *F. caucasica* s.str. (*F. caucasica* 1). Two other groups of haplotypes related to *F. caucasica* 1 are named *F. caucasica* 2 and 3. We have not discovered significant morphological differences between *F. caucasica* 1 and *F. caucasica* 2; therefore, we treat all of the three groups as *F. caucasica* s.l. However, the single specimen that comprises *F. caucasica* 3 was not available for our study, so its morphological similarity with *F. caucasica* 1 and 2 is open to debate. The divergence between the groups within *F. caucasica* s.l. (1.2–2.1% in ITS1-2 and 0.8–1.4% in *trn*L-F) somewhat exceeds the level of variability within other species of *F*. subg. *Trachycolea* (Table 2). Moreover, the Caucasus specimens from Krasnodar Territory, Russia possess ITS1-2, similar to other specimens of *F. caucasica* 1, whereas its *trn*L-F locus is similar to that of the specimens of *F. caucasica* 2. In another Caucasus specimen, collected in the Chechen Republic, Russia, its ITS1-2 is identical to that of *F. caucasica* 1, whereas its *trn*L-F is identical to that of *F. dilatata* (this species is widely distributed in the Caucasus, but is poorly sampled in the current study). Both cases may be due to hybridization between *F. caucasica* 1 and *F. caucasica* 2 (in the first case), and between *F. caucasica* 1 and *F. dilatata* (in the second case). In both events, *F. caucasica* 1 is the male parent, and its ITS1-2 region presents a single copy, which suggests the completeness of concerted evolution in both units. The latter suggests that the hybrids represent long-existing and evolved units.

The well-supported subclade B (98/100/1.00) contains specimens from eastern Siberia and the south of the Russian Far East that are similar to *F. caucasica*, *F. koponenii*, and *F. parvistipula* in the morphology of their weak phases. However, these specimens (especially those from the Primorye Territory) are distinguished by the shape of their well-developed leaves. In general, they are morphologically indistinguishable from the type specimen of *F. conistipula* from Japan, so we treat subclade B as belonging to this species. The specimens of *F. conistipula* demonstrated a low level of sequence variability in their ITS1-2 (0.3%) and were identical in their *trn*L-F. This species is resolved in an unsupported relationship with *F. dilatata* and is also considered to belong to *F*. sect. *Trachycolea*. Both species share similar stylus morphology (rather large and foliaceous). 

The last group of specimens that are morphologically similar to *F. parvistipula* (subclade D) is resolved in clade III (*F*. sect. *Integristipulae* II) and is well-supported (100/100/1.00). This subclade is composed of five specimens from the south of the Russian Far East. Due to their morphological similarity with the original material of *F. fukuzawana* in the shape of the leaf lobule, the subclade is treated here as conspecific with this specimen and is described as a new species. The infraspecific sequence variability in *F. fukuzawana* is 0.1% in ITS1-2, and is absent in *trn*L-F. The weak phases of this species (including the type specimen) have *dilatata*-type morphology, while well-developed plants sometimes bear underleaves that are partially similar to those of species within *F*. sect. *Integristipulae* II, including *F.* ‘*muscicola*’. The finding of *F. fukuzawana* within *F*. sect. *Integristipulae* II restricts the treatment of the morphological range of *F*. sect. *Trachycolea*. 

### 3.3. Other Groups

The specimens that match well with the morphological treatment of *F. koponenii* in Hattori [33] were sequenced from the southern parts of eastern Siberia and the south of the Russian Far East. This species was described from eastern Siberia and is vegetatively similar to *F. parvistipula* (Hattori in Koponen et al. [34]). The sequenced specimens compose a subclade (52/97/1.00) that splits into two grades. However, no correlations have yet been found between this splitting and the morphology, ecology, or geographic distribution of the haplotypes in the two grades. Moreover, the infraspecific variability between these two grades was discovered only for ITS1-2 (1.2%) and has not been found in *trn*L-F (Table 2). Due to the close phylogenetic relationship of the subclade of *F. koponenii* to *F. dilatata* and other species of *F*. sect. *Trachycolea*, *F. koponenii* is confirmed here to belong to this section. Although the perianth ornamentation of this species serves as a diagnostic feature, perianths have not been found in any of the sequenced saxicolous specimens of *F. koponenii* from eastern Siberia. The lack of perianths makes *F. koponenii* difficult to distinguish from *F. parvistipula* and similarly named specimens. 

Three specimens named as *F.* ‘*muscicola*’ and four specimens named as *F. aberrans* from eastern Siberia and the south of the Russian Far East are found in separate subclades (100/100/1.00 and 99/100/1.00, respectively). These subclades are intermingled within the clade of *F*. sect. *Integristipulae* II. The specimens of *F.* ‘*muscicola*’ are morphologically similar to Kamimura’s *F. muscicola* ‘Form A’ [19] in the shape of their leaves, underleaves, and perianths, but differ from the type specimens of *F. muscicola*. The specimens of *F. aberrans* are morphologically similar to the type specimens of *F. aeolotis* var. *aberrans*. Both subclades have low variability in ITS1-2 (0.4% in *F.* ‘*muscicola*’, 0.5% in *F. aberrans*) and lack variability in *trn*L-F. Since *F.* ‘*muscicola*’ and *F. aberrans* are found within *F*. sect. *Integristipulae* II, and the specimens of *F.* ‘*muscicola*’ and the type of *F. muscicola* (as well as the sequenced specimens of *F. aberrans* and the type of *F. aeolotis* var. *aberrans*) share underleaves that are more than 3.5× the stem width (thus diverging from the *dilatata*-type), this further restricts the morphological circumscription of *F*. sect. *Trachycolea*.

## 4. Discussion

### 4.1. Frullania parvistipula

**Figure 2:**
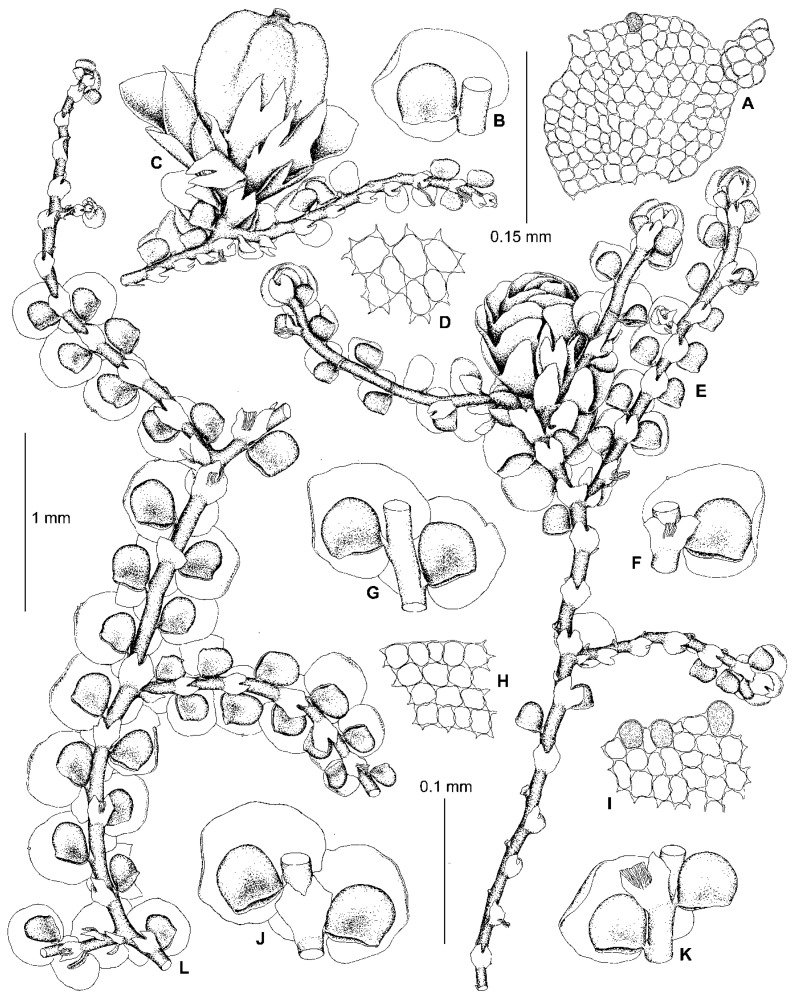
*Frullania parvistipula* Steph.: (**A**)—detached leaf with marginal cells proliferating into a young shoot; (**B**,**F**,**G**,**J**,**K**)—shoot fragments showing leaf lobes, lobules, and underleaves; (**C**)—female shoot; (**D**)—basal leaf cells; (**E**)—male shoot; (**H**,**I**)—marginal leaf cells. Scale bars: 0.1 mm for (**D**,**H**,**I**), 0.15 mm for (**A**), 1 mm for (**B**,**C**,**E**,**F**,**G**,**J**–**L**). (**A**,**H**,**I**) from *Mamontov 182-1-1* (KPABG). (**B**,**F**,**G**,**J**–**L**)—from *Mamontov 737-1-2* (MHA). (**E**) from *Brinda 9978* (MHA).

**Figure 3:**
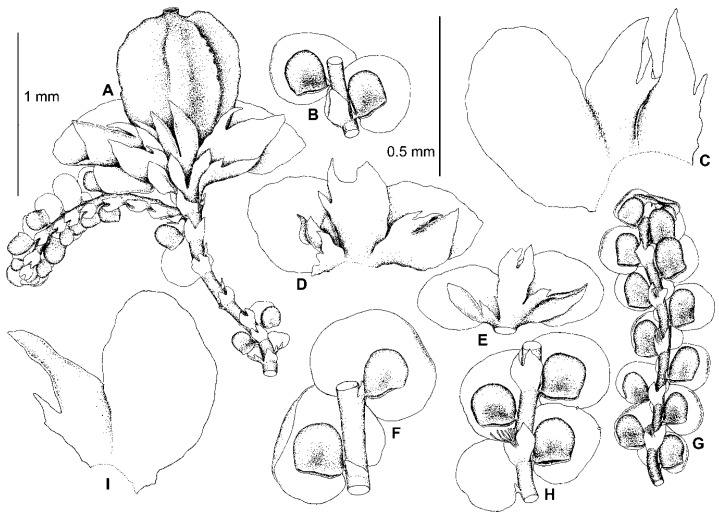
*Frullania parvistipula* Steph.: (**A**)—female shoot. (**B**,**F**–**H**)—shoot fragments showing leaf lobes, lobules, and underleaves. (**C**,**D**,**I**)—innermost female bracts and bracteoles. (**C**)—female bracts and bracteoles—innermost ones (**D**). Scale bars: 0.5 mm for (**C**,**I**), 1 mm for (**A**,**B**,**D**–**H**). (**A**,**D**,**E**) from *Mamontov 716-1-1* (MHA). (**B**,**F**–**H**)—from *Mamontov 737-1-2* (MHA). (**C**,**I**) from *Mamontov 182-1-1* (KPABG).

Kamimura [19] placed *F. parvistipula* in synonymy with *F. muscicola*, regarding it as an environmental modification (‘form C’) of that species [19] (p. 29). Hattori [33], however, resurrected the species and considered it to be distinct, albeit closely related to *F. muscicola.* According to Hattori’s treatment, the morphology of the leaves and underleaves of *F. parvistipula* corresponds to the *dilatata*-type in the sense of Mamontov et al. [11]. Likewise, the specimens sequenced here within subclade C (*F. parvistipula*) have only *dilatata*-type morphology (Figure 2 and Figure 3). As shown in Figure 1, the sequenced specimens of *F.* ‘*muscicola*’ are not related to *F. parvistipula*, so the synonymy and close relationship between *F. muscicola* and *F. parvistipula* is not supported.

**Figure 4:**
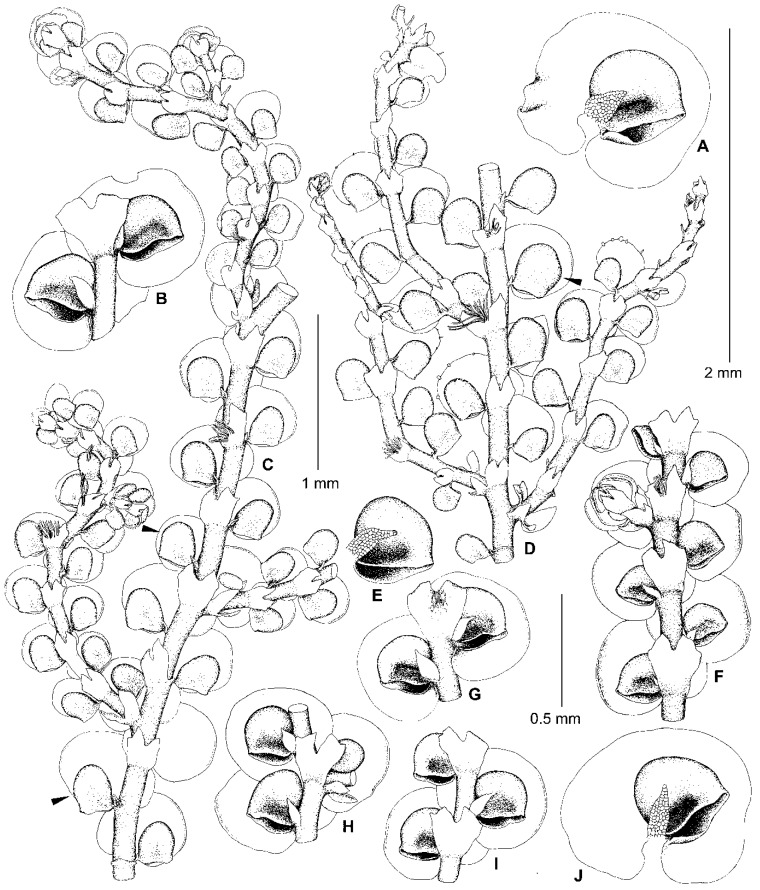
*Frullania conistipula* Steph.: (**A**,**J**)—Isolated leaves with stylus cells indicated. (**B**,**F**–**I**)—Shoot fragments showing leaf lobes, lobules, and underleaves. (**E**)—Leaf lobule. *Frullania parvistipula* Steph.: (**C**,**D**)—Sterile shoots. Scale bars: 0.5 mm for (**A**,**E**,**J**), 1 mm for (**A**,**B**,**F**–**H**), 2 mm for (**C**,**D**). (**A**,**B**,**E**–**I**) from *Mamontov 801-1-9* (MHA). (**C**,**D**)—from lectotype, *Okamura 170* (G00067235).

The original description of *F. parvistipula* [35] briefly characterizes the habit of the species, as well as mostly qualitative features of the leaves, lobules, and underleaves. Based upon an examination of the type specimen, Hattori [32] amended this description by including numerous measurements, as well as a description of the gynoecia. Since the lectotype of *F. parvistipula* (G00067235!) is too old and cannot be sequenced, only morphological data are available to compare *F. parvistipula* with the specimens sequenced here. As shown in Figure 4, the type shoots of *F. parvistipula* are relatively small and have caducous leaves. Furthermore, the majority of the leaf lobules are somewhat too distinctly turned from the stem, widest near the middle or in the lower third, rarely in the upper third, with the mouth straight or truncate, with almost equal mouth valves, sometimes with an ill-defined constriction above the lobule mouth, with the anterior side (the front face between the mouth apex and the lobule tip) characteristically convex to round-angled (Figure 4C,D, arrows). The majority of the studied specimens of subclade C from China, Russia, and the USA consist of relatively small plants (leafy shoots ca. 0.7 mm wide), with caducous leaves and rather small underleaves, and with leaf lobules that are mostly longer than wide and similar to those in the type material of *F. parvistipula.* The male branches and perianths have not been observed by us, nor by Hattori [32] in the lectotype of *F. parvistipula*, although the female inflorescences were described as terminal with two pairs of bracts, the innermost bract lobes being elliptical with obtuse to acute apices and connate with the triangular lanceolate bract lobules for about half their length [32]. In the majority of the sequenced specimens of subclade C, we have found the innermost bract lobes of the female plants to have rounded and obtuse to acute apices. Due to the similarity between the lectotype specimen of *F. parvistipula* and the sequenced specimens of subclade C in the shape of their leaves, underleaves, and female bracts, we consider all of these specimens to belong to *F. parvistipula*. A description of this species based on type material and sequenced specimens is provided in the Section 5. Taxonomy.

### 4.2. Frullania caucasica

This species shows a significant plasticity in the shape of its leaf lobules and styli; the styli sometimes consist of several wide and long cells, which converge with the styli of weak plants of *F. conistipula.* This morphological plasticity makes it extremely difficult to distinguish *F. caucasica* from morphologically similar species such as *F. conistipula* and *F. fukuzawana*, for which mature perianths are still undescribed (as in *F. caucasica*), as well as from sterile specimens of *F. parvistipula.*

The hypothesis of the long existence and evolution of hybrids between *F. caucasica* 1 and *F. caucasica* 2, and between *F. caucasica* 1 and *F. dilatata* (see Section 3.2), may be supported by the fact that androecia have never been found in *F. caucasica*, although unfertilized gynoecia with young perianths are widely distributed. The latter fact suggests that the populations of *F. caucasica* have reproduced only vegetatively for a certain length of time, so the discovered hybrids may have originated before the loss of androecial plants, perhaps before the penetration of *F. caucasica* into North America. Therefore, a narrow distribution of the discovered hybrids may be due to the ancient age of the hybridization events. It should be noted, however, that the discussed hybrids were discovered by accident and only in a few specimens, so the distribution of such hybrids, as well as the distribution of *F. caucasica* 1 and 2, needs separate studies utilizing an expanded sampling of *F. caucasica* specimens.

### 4.3. Frullania conistipula

**Figure 5:**
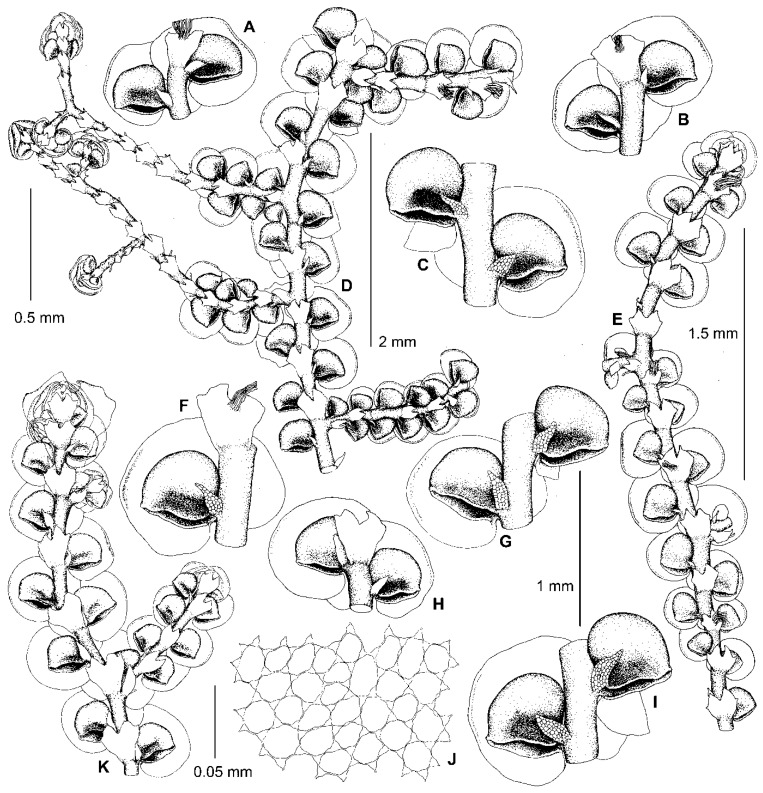
*Frullania conistipula* Steph.: (**A**–**C**,**F**–**I**)—Shoot fragments showing leaf lobes, lobules, and underleaves. (**D**,**E**,**K**)—Sterile shoots. (**J**)—Cells of the leaf base. Scale bars: 0.05 mm for (**J**), 0.5 mm for (**C**,**F**,**G**,**I**), 1 mm for (**A**,**B**,**H**), 1.5 mm for (**E**), 2 mm for (**D**,**K**). (**A**–**D**,**F**–**I**) from type, *Faurie 135* (G00069157). (**E**) from *Bakalin K-37-5-06* (KPABG). (**J**,**K**) from *Mamontov 801-1-9* (MHA).

**Figure 6:**
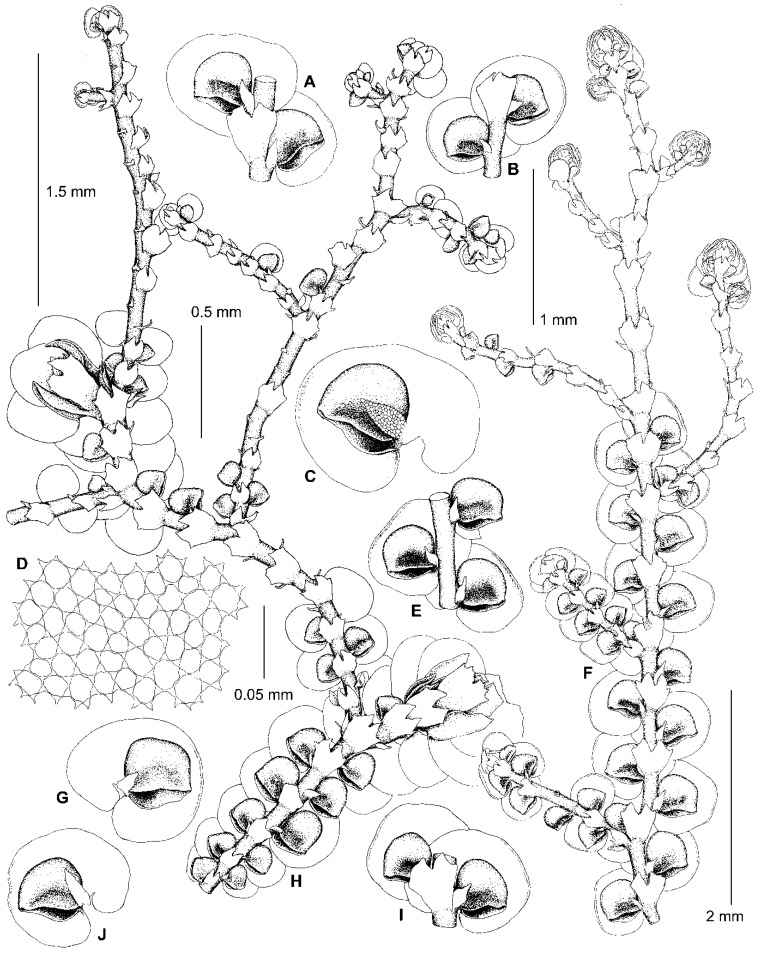
*Frullania conistipula* Steph.: (**A**–**C**,**E**,**G**,**I**,**J**)—Shoot fragments showing leaf lobes, lobules, and underleaves. (**D**)—Midleaf cells. (**F**)—Sterile shoot. (**H**)—Female shoot. Scale bars: 0.05 mm for (**D**), 0.5 mm for (**C**), 1 mm for (**A**,**B**,**E**,**G**,**I**,**J**), 1.5 mm for (**H**), 2 mm for (**F**). (**A**,**C**,**D**–**G**,**J**,**I**) from *Mamontov 801-1-9* (MHA). (**B**,**H**) from *Bakalin K-37-5-06* (KPABG).

We have found that the morphology of the type material of *F. conistipula* (Figure 5A–D,F–I), a species described from Northern Honshu, Japan and synonymized with *F. muscicola* var. *inuena* (Steph.) Kamim. [19] (p. 24), matches well with the morphology of specimens within subclade B (Figure 4A,B,E–J, Figure 5E,J,K and Figure 6). We therefore consider specimens in subclade B to belong to *F. conistipula.* The synonymization of this species with *F. inuena* Steph. is not accepted here, due to morphological differences in the shape of styli and underleaves between the lectotypes of *F. conistipula* (G00069157!) and *F. inuena* (G00066750!). Indeed, in the lectotype specimen of *F. conistipula*, the styli are foliaceous and 9–14 cells long by 5–9 cells wide, while the underleaves are up to 2.7× the stem width, and are often dentate in their upper third, with straight or concave margins in their lower half. By comparison, the lectotype specimen of *F. inuena* has styli that are filiform to triangular and 3–5 cells long by 2–3 cells wide at the base, as well as underleaves that are 2.9–3.2× the stem width and have entire margins which are convex in their lower half. Due to the shape of the underleaves, which is divergent from the *dilatata*-type, *F. inuena* is better placed within *F*. sect. *Integristipulae* II. The reduction of *F. inuena* to a variety or a synonym of *F. muscicola* is not accepted here due to morphological differences between the type specimens of both names. The discussion on this taxonomic issue is expected in a separate future study of the *F. muscicola* complex. The original description of *F. conistipula* in Stephani [35] briefly characterizes the habit of the species and includes mostly quantitative features of the leaf lobes, lobules, and underleaves. An amended description of this species based on its lectotype specimen, as well as sequenced specimens (subclade B), is provided in the Section 5. Taxonomy.

### 4.4. Frullania fukuzawana

**Figure 7:**
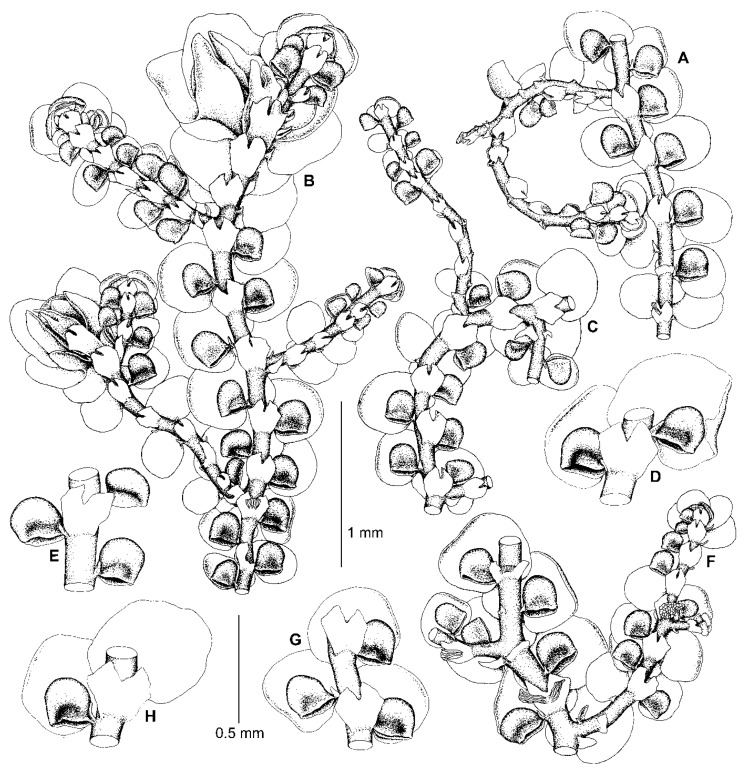
*Frullania fukuzawana* Steph. ex Mamontov, J. J. Atwood & Vilnet: (**A**,**C**,**D**–**H**)—Shoot fragments showing leaf lobes, lobules, and underleaves. (**B**)—Female shoot. Scale bars: 0.5 mm for (**D**,**E**,**G**,**H**), 1 mm for (**A**–**C**,**F**). All from holotype, *Okamura 422* (G00066779).

*Frullania fukuzawana* is an invalid name due to it being published without a formal description. The name is based upon a specimen that was collected by Shûtai Okamura in Fukuzawa, Japan in 1913, and cited by Bonner [36] (p. 307) as a “Nomen herbariorum?”. In studying the original material deposited in the G herbarium, Hattori [37] (p. 208) gave a brief description of the plants, although he did not formally accept the species, and placed *F. fukuzawana*, along with “Form C” of *F. muscicola* [19] (p. 29, Figure 6: 24–47), as tentative synonyms of *F. muscicola*, while nevertheless also stating that *F. fukuzawana* “may probably be a different species”. We have found that the G plants are similar to weak phases of *F. conistipula* in regard to the shape of their leaf lobes, lobules, and underleaves, as well as in the presence of caducous leaves. Indeed, the leaf lobes in the original material of *F. fukuzawana* are rounded at the apex and the leaf lobules are mostly as long as they are wide, and rarely slightly longer than wide or wider than long, with no traces of a beak and usually with distinctly unequal mouth valves, of which the dorsal valve is longer than the ventral one. The bract lobes in the specimen are rounded at the apex, while the underleaves are up to 2.3 times wider than the stem in all studied shoots, divided ca. 1/3 of their length, narrowed to the base, and not auriculate. However, typical expressions of *F. conistipula* have shoots with frequent caducous leaves and *Bazzania*-like flagelliform branches (see circumscription of *F*. sect. *Trachycolea* below), auriculate lobe bases, mostly conical (widest in lower third) leaf lobules without a constriction of the mouth, and usually foliaceous styli. By comparison, the shoots of *F. fukuzawana* are more persistently leaved, or the leaves have fallen off in several places along the shoot, giving the shoots a discontinuous foliation. Furthermore, the lobe bases are not as dilated, the largest leaf lobules are sometimes distinctly inflated in the upper third and have clearly constricted mouths, and the styli are significantly shorter and usually subulate. *Frullania fukuzawana* is here validated as a distinct species, given the numerous morphological differences with other species in the *F. parvistipula* complex. A description and illustration (Figure 7) of this species, based on the original specimen (G00069157!) cited in Bonner [36], is provided in the Section 5. Taxonomy. We have sequenced five specimens from the Primorye Territory and Sakhalin Region of Russia, which are indistinguishable from *F. fukuzawana* in the shape of leaf lobules, styli, and underleaves, and in the presence of caducous leaves. The plants from the sequenced specimens, however, are larger in size and seem to represent well-developed phases of this species. A description and illustrations of the new specimens are expected in a separate future study devoted to discussing the distinctions between *F. caucasica*, *F. fukuzawana*, and *F. koponenii*.

### 4.5. Frullania muscicola

The specimens sequenced here as *F.* ‘*muscicola*’ (Figure 1) from eastern Siberia and the Russian Far East are morphologically identical with specimens distributed as *Frullania muscicola*, No. 127, in the Hepaticae Japonicae, Ser. 3 (1950) exsiccatae [38]. In our opinion, these specimens are morphologically closely related to the type specimen of *F. muscicola* from Yunnan Province, China (G00067208!). However, they differ in the shape of their leaf lobules and underleaves, and so are not conspecific with it. Moreover, we did not see any specimens from Asia (including Russia, continental China, Taiwan, Japan, and the Korean Peninsula) that we would strictly consider to be conspecific with the type of *F. muscicola*. Therefore, the latter species is possibly endemic to Yunnan or the Himalaya area, like numerous other East Asia *Frullania* endemics. A detailed morphological comparison between the type of *F. muscicola* and the sequenced specimens of *F.* ‘*muscicola*’, as well as the description of the latter as a new species, is expected in a separate future study.

### 4.6. Frullania aeolotis var. aberrans

The taxon *F. aeolotis* var. *aberrans* was described as being from the Shaanxi Province of China but was later synonymized with *F. subdilatata* and included in the *F. dilatata* complex by Hattori [39]. However, later, it was synonymyzed with *F. muscicola* [40] and *F. riparia* [41]. Furthermore, the type variety of *F. aeolotis* Mont. & Nees, *F. aeolotis* var. *aeolotis*, has been considered to be a synonym of *F. riparia* by Schuster [15] and a synonym of *F. ericoides* by Schumacker & Váňa [41]. The placement of *F. aeolotis* in synonymy with *F. ericoides* seems justified considering that “perianthio obovato truncato mucronato plano-compresso subtus carinato, margine carinaque muriculatis” is noted in the protologue of *F. aeolotis* [42] (p. 461). By comparison, mature perianths are unknown in *F. riparia.* Furthermore, Montagne places *Jungermannia squarrosa* Nees and *J. ericoides* Nees in synonymy with *F. aeolotis.*

Here, we consider the sequenced specimens of *F. aberrans* to be conspecific with isolectotypes of *F. aeolotis* var. *aberrans*, preserved in G (G00113360!, G00113361!) and LE(!). These syntypes and the sequenced specimens share similarly shaped leaves and underleaves, as well as lobules that are longer than wide, inclined from the stem at an angle of up to 45°, and have a small beak. As shown in Figure 1, the sequenced specimens of *F. aberrans* are not conspecific with *F.* ‘*muscicola*’, *F. riparia*, or *F. ericoides*, and thus represent a separate species. Below, we provide the necessary combination for this species (see the Section 5. Taxonomy).

Within *F*. subg. *Trachycolea*, the species *F. crispiplicata* Yuzawa & S. Hatt., in our opinion, is most similar to *F. aberrans* in its superficial appearance. However, in our opinion, *F. aberrans* is not conspecific with *F. crispiplicata*, in that the shape of the leaf lobules is different from the holotype of the latter species (NICH-237407!). *Frullania crispiplicata* has never been sequenced, so its possible relationship with *F. aberrans* remains open to debate. In the protologue of *F. crispiplicata*, Yuzawa and Hattori [43] note the species to be closely related to *F. pedicellata* Steph., although differing in the shape of the perianth. *Frullania crispiplicata* further differs, in our opinion, from the lectotype of *F. pedicellata* (G00067240!) in the shape of the leaf lobules. A detailed discussion on *F. aberrans*, including descriptions and illustrations of the type material and the sequenced specimens, along with a discussion on its distribution, ecology and the morphological distinctions between *F. subdilatata*, *F. crispiplicata*, *F. kochiensis* Steph., and *F. pedicellata*, is expected in a separate future study.

### 4.7. Frullania dilatata subsp. asiatica and F. subdilatata

**Figure 8:**
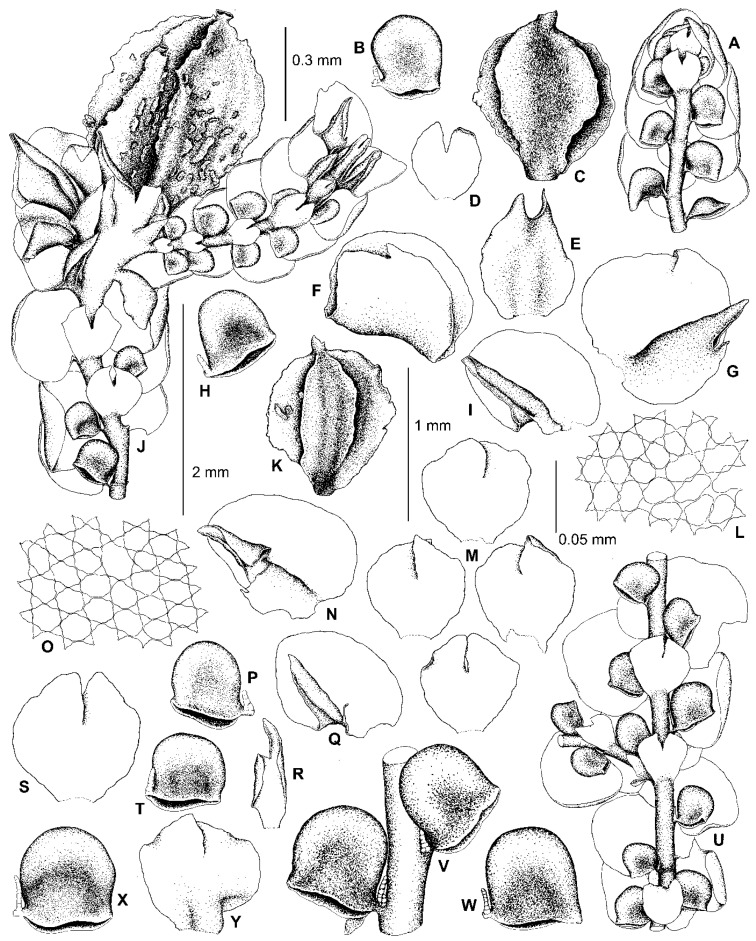
*Frullania asiatica* (S.Hatt.) Mamontov & J. J. Atwood: (**A**,**U**)—Shoot fragments showing leaf lobes, lobules, and underleaves. (**B**,**H**,**P**,**T**,**V**,**W**,**X**)—Leaf lobules with styli. (**C**,**K**)—Perianths. (**D**,**M**,**S**,**Y**)—Underleaves. (**E**,**R**)—Female bracteoles. (**F**,**G**,**I**,**N**,**Q**)—Female bracts. (**J**)—Female shoot. (**L**)—Cells of leaf lobe base. (**O**)—Cells of leaf lobe middle. Scale bars: 0.05 mm for (**L**,**O**), 0.3 mm for (**B**,**H**,**P**,**T**,**V**,**W**,**X**), 1 mm for (**A**,**C**,**D**,**E**,**F**,**G**,**I**,**K**,**M**,**N**,**Q**,**R**,**S**,**Y**), 2 mm for (**J**,**U**). All from holotype, *Iwatsuki*, *Sharp & Sharp B145a* (NICH-262371).

Hattori [39] described *F. dilatata* subsp. *asiatica* based on differences from *F. dilatata* in the shape of the underleaves, which are “slightly wide and more often without lateral teeth or angles” (l.c.), as well as the shape of the styli, which are “minute, composed of a row of about 4–5 uniseriate cells (or often two cells wide at the very base)” (l.c.). It should be noted that the underleaves in the holotype of *F. dilatata* subsp. *asiatica* (NICH-262371!) are much broader than in all other species of *F*. sect. *Trachycolea* if one compares the ratios of the *underleaf width/stem width*. Indeed, in *F. dilatata* subsp. *asiatica*, this ratio varies in the range of (2.5–)3.5–3.9, whereas in other species of *F*. sect. *Trachycolea*, including *F. dilatata*, this ratio usually is about 2.0–2.7. In *F. dilatata*, however, the styli are foliaceous, 3–9 cells wide at the base in European plants [17,39], as well as in the sequenced specimens from the Altai Mts. Although the shoots of *F. dilatata* subsp. *asiatica* from the type specimen are rather large, up to 2.2 mm wide in vegetative sectors (vs. up to 1.5 mm wide in *F. dilatata*), the styli are consistently filiform throughout (Figure 8). Some leaf lobules in *F. dilatata* subsp. *asiatica* are of the *dilatata* type, i.e., nearly as long as wide, conical or subquadrate in outline, widest near the middle or in the lower third (Figure 8F,H), and are similar to the leaf lobules of several species of *F*. sect. *Trachycolea* (including *F. appalachiana*, *F. azorica*, *F. caucasica*, *F. conistipula*, *F. dilatata,* and *F. virginica*) and *F*. subg. *Frullaniopsis* (including *F. austinii*, *F. oakesiana*, *F. stylifera,* and *F. takayuensis*). Other leaf lobules in *F. dilatata* subsp. *asiatica* are longer than wide, widest in the upper third, with inflated postero-upper parts (Figure 8A), and more closely resemble species of *F*. sect. *Intergristipulae* II, where numerous species (including *F. davurica*, *F. hamatiloba* Steph., *F. kagoshimensis* Steph., *F. pedicellata*, *F. taradakensis,* and *F. usamiensis* Steph.) have leaf lobules with an inflated postero-upper part. We think that the best explanation for the morphological differences in the shape of leaf lobules, styli, and underleaves between *F. dilatata* from Europe and the Altai Mts. plants versus the India *F. dilatata* subsp. *asiatica* is that the Indian plants belong to a separate species, *F. asiatica* comb. et stat. nov. The necessary combination for this taxon is provided in the Section 5. Taxonomy. The rather large shoots with small filiform styli of *F. asiatica* also make this species similar to the species of *F*. sect. *Intergristipulae* II, including *F. davurica*, *F. jackii*, *F. muscicola*, *F. riparia*, *F. taradakensis*, and many others. Therefore, we consider *F. asiatica* to belong to *F*. sect. *Intergristipulae* II, rather than *F*. sect. *Trachycolea*, due to characteristics of the leaf lobules, styli, and underleaves.

Hattori [39] considered *F. subdilatata* a member of the *F. dilatata* complex, and the phylogenetic affinity of this species still remains unresolved due to the absence of its DNA sequences. However, underleaves in the type plants of this species deviate from the *dilatata*-type, being entire-margined and only shallowly bifid at the apex (to 1/8–1/10 of the underleaf length), with lateral margins that are often narrowly to widely recurved. Due to the shallowly bifid underleaves, *F. subdilatata* is partly similar to the type plants of *F. muscicola,* as well as several species (*F. kagoshimensis*, *F. taradakensis*, and *F. usamiensis*) that are confirmed to belong to *F*. sect. *Intergristipulae* II [10]. Therewith, the leaf lobules in the type plants of *F. subdilatata* are mostly of the *dilatata* type (as in *F. caucasica*), but are usually clearly longer than wide, sometimes widest in the upper third and with an inflated postero-upper part (as in *F. asiatica*), and slightly beaked (as in *F. aberrans*). The styli are subulate to foliaceus (although Hattori [37] (p. 220) described them as “small, filiform”), the perianth is strongly trigonous, elliptic to widely obovate (as are those illustrated for *F. ocumiensis* Steph. in Kamimura [19]), with a subtruncate to truncate apex and a small beak (as in *F. kagoshimensis* var. *minor* Kamim. and *F. hamatiloba*, see Kamimura [19]), and the perianth surface is partly similar to that of *F. asiatica* (Figure 8A) in bearing multicellular tubercles and laciniae (in *F. subdilatata,* these outgrowths occur mostly on the perianth keels, though they are also scattered over the surface). Due to the combination of these characteristics of the leaves, underleaves, and perianths, we attribute *F. subdilatata* to *F*. sect. *Intergristipulae* II. 

### 4.8. Frullania fuegiana, F. semivillosa, and F. tibetica

*Frullania fuegiana* is known only from southern Patagonia (in both Argentina and Chile) [44]. In the protologue of this species [35] (p. 428), the leaf lobules are characterized as being almost twice as long as wide, with a stylus that is foliaceous, narrowly lingulate, and almost as long as the lobule. Moreover, the underleaves are described as reniform, up to 3× as wide as long, and with entire margins and widely triangular lobules, which are rounded at the apices. Such lobule and underleaf morphologies differ from the *dilatata*-type, and is more similar to those of *F. fertilis* De Not., a widely distributed Patagonian species that is molecularly placed within *F*. subg. *Microfrullania* (R. M. Schust.) R. M. Schust. Due to its similarity with *F. fertilis* in the shape and arrangement of leaf lobules, in the shape and size of the styli, and in the development of the perianths on abbreviated lateral branches, *F. fuegiana* is provisionally attributed here to *F*. subg. *Microfrullania*.

*Frullania semivillosa* Lindenb. & Gottsche was described from Mexico and has recently been attributed to Brazil [45]. According to the description of *F. semivillosa* in Lima [45], this dioicous species has leaf lobules and underleaves that seem to be of the *dilatata* type, i.e., the leaf lobules are galeate, with the opening dilated or undilated and lacking a beak, and the underleaves are isodiametric, ca. 2× stem width, obconical, and with a tooth on each side. However, *F. semivillosa* also has leaf lobules that are 300–400 µm long and 110–380 µm wide, i.e., 1.05 to 2.72 times longer than wide. Such elongated leaf lobules with dilated mouths are unknown in the sequenced representatives of *F*. sect. *Trachycolea* but are characteristic of *F*. sect. *Australes* species [46] (pp. 524–526, Figures 5–7). The perianth ornamentation is described by Lima [45] as covered with short to long laciniae, reflecting the specific epithet of this species. This characteristic also distinguishes *F. semivillosa* from the sequenced members of *F*. sect. *Trachycolea*, where the perianth surface varies from smooth to tuberculate, papillate, or asperate [15] (pp. 148, 153), [17] (pp. 475, 476). The laciniate perianth surface of *F. semivillosa* is more similar to that of *F. glomerata* (Lehm. & Lindenb.) Mont., a South American species with perianth that also bears multicellular laciniae [47]. Sequenced specimens of *F. glomerata* place that species within *F*. sect. *Australes*, and we believe that due to their morphological similarity, *F. semivillosa* should be attributed to this section as well.

*Frullania tibetica* was described as similar to *F. parvistipula* due to its *dilatata*-type vegetative morphology and the presence of asexual reproduction via caducous leaves [18]. Although this species has not been sequenced, the perianths in *F. tibetica* are also covered with spinose outgrowths (like *F. semivillosa*), the largest of which are 6 to 9 cells long. This characteristic contradicts that of the sequenced members of *F.* sect. *Trachycolea* since they possess only rather low tuberculae on the perianth surface. Due to this morphological difference, the taxonomic position of *F. tibetica* remains unresolved between *F.* sect. *Trachycolea* and *F*. sect. *Integristipulae* II until specimens of this species can be included in a molecular study.

### 4.9. The Circumscription of Frullania sect. Trachycolea

The autonym *F.* subg. *Frullania* has been used as a replacement name for both *F.* subg. *Trachycolea* Spruce and *F.* subg. *Thyopsiella* Spruce, depending on which of these subgenera is interpreted to include the type species. When Raddi [48] described *Frullania*, he simultaneously described two superfluous species, *F. major* and *F. minor*, both containing older names in their synonymy. Raddi [48] included *Jungermannia dilatata* L. (≡*F. dilatata* (L.) Dumort.) under *F. major* Raddi, whereas *J. tamarisci* L. (≡*F. tamarisci* (L.) Dumort.) was cited under *F. minor* Raddi. The earliest lectotypification of *Frullania* was made by Evans [49] (p. 468), who designated *F. dilatata* as the type of the genus. Most typifications made by Evans are now considered to be mechanical because he often selected the first species listed as the type (see Canon 15 of the American Code of Botanical Nomenclature [50]). Under Art. 10.6 of the current Code [51], mechanical typifications such as these can be superseded. However, Hentschel et al. [52] argued that Evans’s selection of *F. dilatata* was not mechanical since he selected the older name instead of *F. major*, the first name listed. Since *F. dilatata* is also the type of *F.* subg. *Trachycolea*, that subgenus was replaced by the autonym, *F.* subg. *Frullania*. Lima et al. [53] have more recently argued that Evans´ lectotypification was nevertheless still a mechanical selection and that the name *F.* subg. *Trachycolea* should be reinstated. Indeed, on account of Alexander W. Evans being a signatory of the American Code [see list of signatories in McNeil et al. [54] (p. 1446)], his 1918 publication incontrovertibly adopts a mechanical method of type selection based on Art. 10.7C [50]. The next earliest lectotypification of *Frullania* was made by Frye & Clark [55] (p. 736) in designating *F. tamarisci* as the type of the genus. Since *F. tamarisci* is also the type of *F.* subg. *Thyopsiella*, that subgenus is replaced by the autonym *F.* subg. *Frullania*.

According to the obtained molecular and morphological data, *F.* sect. *Trachycolea* is considered here to include the following eight sequenced species: *F. appalachiana*, *F. azorica*, *F. caucasica*, *F. conistipula*, *F. dilatata*, *F. eboracensis*, *F. koponenii*, and *F. virginica*. Compared to the other sections within *F.* subg. *Trachycolea*, this section is morphologically characterized by the following combination of features: (1) dioicous sexuality; (2) fundamentally trigonous perianths, never pluricarinate, with a smooth or tuberculate surface, and with the perianth beak never clearly elongated compared to the body of the perianth; (3) intermediate thickenings always present in the leaf lobe cells; (4) underleaves always with a narrow base, without appendages; (5) a (1.1–)1.5–2.7 ratio of the *underleaf width/stem width*; (6) well-developed leaf lobules that are largely conical to subquadrate, more or less subisodiametric, rarely somewhat longer than wide, not beaked, with the upper part of the lobule never strongly inflated (sometimes rather inflated in *F. caucasica*), the lobules are not or weakly constricted above the mouth, without a protruding rostral portion (characteristic of some species of *F*. sect. *Australes* and *F*. sect. *Integristipulae* II), the lobule mouth is usually not constricted (except some phenotypes of *F. caucasica*), so that the mouth width is only slightly smaller or equal or larger than the width of the lobule; (7) the valves of the leaf lobule mouth are unequal, with the dorsal valve always larger than the ventral one, but without a triangular laminal portion; (8) styli are subulate to lanceolate or foliaceous.

One of the characteristic features occurring in some species of this section is the *Bazzania*-like leafless flagellae (flagelliform shoots) resulting from asexual reproduction by caducous leaves. Such flagellae are developed in *Bazzania nudicaulis* A.Evans, *B. bidentula* (Steph.) W.E. Nicholson, *B. parabidentula* Bakalin, and *B. denudata* (Lindenb. & Gottsche) Trevis. [56] (p. 80, Figure 97: 3), [57] (Figure 1: 2, 15; Figure 5: 1). Hattori [33] (p. 159) characterized these flagellae in *F. koponenii* as “stolon-like shoots more slender than normal, leafy shoots, not limited in elongation and often again turning to the normal, leafy shoots and often floriferous”. Such flagellae have the same color as the leafy parts of the shoot and usually do not turn blackish brown with age. These *Bazzania*-like leafless flagellae are characteristic of not only *F. koponenii* but also *F. conistipula* (Figure 5D and Figure 6F,H) and *F. appalachiana* [15], (p. 152, Figure 796: 1), where these flagellae sometimes (in *F. koponenii*) stand upwards, away from the substrate. Somewhat similar leafless flagelliform axes occur in *F. eboracensis* and *F. caucasica*, as well as in *F. fukuzawana* of *F.* sect. Integristipulae II, *F. parvistipula* of *F.* sect. Australes, and in *F. tibetica* [18] (p. 307, Figure 1I). However, in the latter five species, the caducous leaves (or only caducous lobes or caducous lobules) have usually fallen off in several parts along the shoot, thus forming peculiar discontinuous foliation resulting in an alternation of foliated and defoliated parts of the shoot (Figure 2C,E and Figure 7B), see also [15] (p. 135, Figure 793: 9). In *F. bolanderi*, a species that has the superficial appearance of *F. koponenii* but belongs to *F*. subg. *Frullaniopsis* [11], Hattori [33] (p. 159) noted a second type of leafless flagellae: “stolon-like shoots robust, equally thick as the normal, leafy shoots, short and limited in elongation”. The flagellae of this second type are also characteristic of one more species of *F*. subg. *Frullaniopsis*, *F. austinii* J.J.Atwood, Vilnet, Mamontov & Konstant. [11], but also of several species of the genus *Acrolejeunea*, including *A. emergens* (Mitt.) Steph., *A. heterophylla* (A. Evans) Grolle & Gradst., *A. pusilla* (Steph.) Grolle & Gradst., *A. recurvata* Gradst., and *A. torulosa* (Lehm. & Lindenb.) Schiffn., which bear similar flagelliform axes [58] (Figures VI: 1, VII: 1, VIII: 1, IX: 9), [59] (p. 795, Figure 657: 5). These ‘*Acrolejeunea*’-type flagellae are described in detail by Gradstein [58] (pp. 32–34), and several characteristics common to both species of *Acrolejeunea* and *F.* subg. *Frullaniopsis* can be pointed out. When fresh, the flagellae stand upwards, away from the substrate. They turn brownish with age and are normally short and unbranched (but sometimes are branched), clustered near the apex of the stems or branches, with the remaining underleaves smaller than the normal underleaves and densely arranged (imbricated or squarrose) due to the very short internodes. In *F.* sect. *Trachycolea* species, the underleaves remaining on the flagellae may be nearly squarrose (in *F. koponenii*) and are somewhat smaller than the underleaves of the leafy parts of the shoot (Figure 5D and Figure 6F,H), see also [15] (p. 152, Figure 796: 1). However, the flagellae themselves have normal (non-shortened) internodes, the length of which is comparable with that of the leafy parts of the shoot. The presence of *Bazzania*-like flagellae, thus, distinguishes species of *F.* sect. *Trachycolea* from the similar dioicous species of *F*. subg. *Frullaniopsis.*

In the context of distinctions between *F.* sect. *Trachycolea* and species of other groups, including *F.* sect. *Australes*, *F.* sect. *Integristipulae* II, *F*. sect. *Inflatae*, and *F*. subg. *Frullaniopsis*, the *dilatata*-type leaf lobules characteristic of all species of *F.* sect. *Trachycolea* are considered particularly challenging for discerning species affiliations. The development of this lobule type is discussed in Mamontov et al. [11], where it is hypothesized that *dilatata*-type lobules are a neothenic feature, which, together with *dilatata*-type underleaves (rather small, bifid, narrowed to the base, and not auriculate), result from the origin and/or current distribution of species in extreme (rather dry or continental) habitats. Similarly shaped leaf lobules and underleaves are also found in several groups of *F*. subg. *Chonanthelia* and *F*. subg. *Trachycolea*; however, they are being manifested in different species of these subgenera mainly independently from their phylogenetic relationships and sectional affiliation. The sequenced representatives of *F.* sect. *Trachycolea* (as well as species of *F.* subg. *Frullaniopsis*) bear only this type of lobules and underleaves. Moreover, no *Frullania* species (with or without leaf lobules and underleaves of the *dilatata* type) or species occurring outside of the Holarctic have been found to belong to *F.* sect. *Trachycolea* using molecular markers. Among the sequenced *Frullania*, including those distributed in rather humid areas of Japan, the Kuriles Islands, and eastern North America, only species with *dilatata*-type morphology belong to *F.* sect. *Trachycolea*. In fact, the southernmost localities of species of *F.* sect. *Trachycolea* are in the subtropics of North America (the records of *F. eboracensis* and *F. virginica* in Georgia, Louisiana, and Texas) and Macaronesia (*F. azorica*). However, there are no confirmed records of species of this section from the subtropics of Asia, as in fact, the southernmost localities of *F. conistipula* and *F. koponenii* are only in Japan’s Northern Honshu and Hokkaido, respectively. Due to these circumstances, it can be concluded that: (1) the crown group of this section is relatively young and perhaps is strictly Holarctic (similarly to *F.* subg. *Frullaniopsis*); and (2) the morphological treatment of *F.* sect. *Trachycolea* based on the sequenced members of the section can be used for the presumptive assignment of the Holarctic species to this group. However, it should be noted that some Australasian *Frullania*, namely *F. cranialis* (Hook. & Taylor) Taylor, *F. pentapleura* Taylor, and *F. probosciphora* Taylor, also reveal *dilatata*-type leaf lobules and underleaves [60] (Figures 52 and 62), [61] (Figures 4–6), similar to those in *F.* sect. *Trachycolea* taxa. The latter three species, however, have not been sequenced, so their phylogenetic relationships remain open to debate. Both *F. pentapleura* and *F. proboscifora* are considered to belong to *F.* sect. *Australes* in Söderström et al. [13], while the Australasian (or European?) origin of the sole known specimen of *F. cranialis* will remain questionable until a re-collection is conducted in Western Australia, particularly at or near King George´s Sound, from where this species was described [60] (p. 140). As stressed before, among the Holarctic representatives of other sections of *F.* subg. *Trachycolea*, including *F.* sect. *Australes* and *F.* sect. *Integristipulae* II, ‘*dilatata*-type leaf lobules’ and ‘*dilatata*-type underleaves’ are being manifested in different species mainly independently from each other. Usually, this allows for the distinguishing of species with *dilatata*-type lobules (for example, *F. jackii* Gottsche, *F.* ‘*muscicola*’, *F. pariharii* S.Hatt. & Thaithong, and *F. subdilatata*) by the shape of the underleaves, or vice versa, where distinguishing the species with *dilatata*-type underleaves (*F. ignatovii* Sofronova, Mamontov & Potemkin) can be achieved by the shape of the leaf lobules. Rarely are both leaf lobules and underleaves of the *dilatata*-type found in the same species, making such taxa particularly difficult to distinguish from species of *F.* sect. *Trachycolea.* One of these taxa is *F. parvistipula*, the distinctions of which are discussed below (see the Key and the Section 5. Taxonomy). Another two taxa, with both *dilatata*-type leaf lobules and underleaves, are *F. fukuzawana* and *F. tibetica*. As stressed before (Section 4.8), the taxonomic position of *F. tibetica* remains unresolved between *F.* sect. *Trachycolea* and *F*. sect. *Integristipulae* II until a molecular study of this species is performed. Moreover, the morphological variation of this species is poorly known and needs further study. 

### 4.10. Dichotomous Key to the Species of Frullania sect. Trachycolea and the F. dilalata and F. parvistipula Complexes

The key includes all sequenced species of *F.* sect. *Trachycolea* and some Holarctic species formerly considered to be related, namely *F. aberrans*, *F. asiatica*, *F. fukuzawana*, *F. muscicola* s.str. (sensu Hattori [37]), *F.* ‘*muscicola’*, *F. parvistipula*, *F. subdilata*, and *F. tibetica*.

1. Plants with persistent leaves …………………………………………….……………. 2 

-. Plants with caducous leaves ……………………………………………….…………... 10

2. Underleaves normally more than three times wider than stem. Styli filiform …… 3

-. Underleaves normally up to 2.7× as wide as the stem. Styli subulate or lanceolate or foliaceous ….………………………………………………………………………………….. 6

3. Lobule mouth nodding towards stem at an angle of up to 45° *………… F. aberrans*

-. Lobule mouth perpendicular to stem or inclined away from it …………………..... 4

4. Lobules campanulate, with distinct beak; underleaves bilobed ca. 0.2 of the length …………………………………………………………… *F. muscicola s.str.* (sensu Hattori [37])

-. Lobules galeate, without beak or with small indistinct beak ………….…………… 5 

5. Lobules sub-quadrate, sub-isodiametric to longer than wide, inflated in upper half; underleaves bilobed 0.39–0.41 of the length, entire margined or with a round to acute angulation on one or both sides. Perianths tuberculate ……………………………. *F. asiatica*

*-.* Lobules largely conical to triangular (strongly similar to well-developed lobules of *F. austinii* and *F. conistipula*), mostly wider than long, rarely sub-isodiametric, mostly not inflated in upper half, widest at mouth or near the middle; underleaves bilobed ca. 0.25 of the length, in upper half normally with 1–2 acute or blunt teeth on both sides. Perianths smooth to slightly tuberous …………………………………………………........ *F.* ‘*muscicola*’

6. Perianth ornamentation smooth or mostly so ………………………….. *F. eboracensis*

-. Perianth ornamentation conspicuously tuberculate ……………………………….... 7

7. Gynoecial bracts strongly squarrose when wet, mouth of perianth beak flaring ………………………………………………………………………………………..... *F. virginica*

-. Gynoecial bracts plane when wet, mouth of perianth beak not flaring ………...…. 8

8. Underleaves bilobed 0.10–0.13 of the length, the lateral margins entire, often narrowly to widely recurved. Leaf lobules clearly longer than wide, rarely subisodiametric, mostly widest in upper half, often slightly beaked ……………………………... *F. subdilatata*

-. Underleaves bilobed ca. 0.33 of the length, the lateral margins plane, generally with 1(2) teeth. Leaf lobules as long as or slightly longer than wide, or wider than long, mostly widest near the mouth., mostly not beaked ……………………………………………………9

9. Perianth usually with two ventral keels (though sometimes seeming as trigonous). The largest and most well-developed leaf lobules isodiametric, slightly longer than wide, or wider than long, subquadrate to semicircular or conical, with strongly unequal mouth valves (so that the dorsal valve is sometimes even longer than that is illustrated here for *F. conistipula* in Figure 6G). Styli mostly widest near the base, subulate to narrowly but long (up to 0.75 of the lobule length) lanceolate ………………….………………... *F. azorica*

*-.* Perianth with one sharp ventral keel. Leaf lobules mostly isodiametric or nearly so, with less unequal mouth valves (greatly similar to those illustrated here for *F. conistipula* in Figure 4, Figure 5 and Figure 6). Styli usually more wide, lanceolate to foliaceous, sometimes to often widest near the middle ………………...…………………………………….…. *F. dilatata*

10. Caducous leaves restricted to specialized flagellae; the flagellae mostly erect or incurved back to the dorsal side of the shoot; very rarely caducous leaves absent (in some specimens from Kunashir). Styli mostly filiform or subulate or triangular. Fertilized gynoecia and perianths often present. Usually on trees, but in larch forests in Eastern Siberia (mostly in Transbaikalia) sometimes purely on rocks …………………………… *F. koponenii*

-. Caducous leaves occurring on vegetative stems and/or branches; the parts of the shoots with caducous leaves mostly not erect or incurved back to dorsal side of the shoot …………………………………………………………………………………………………… 11

11. Caducous leaves (both lobes and lobules) mostly occurring on flagelliform shoot apices and lateral branches, but caducous lobules also usually abundant in different parts of shoots. Well-developed leaf lobules mostly conical, mostly widest at mouth, with strongly unequal mouth valves. Styli foliaceous (at least one or some per shoot), up to 9 cells wide at base and to 20 cells long. Fertilized gynoecia unknown. Usually on rocks, only exceptionally on soil on tree base (in Transbaikalia) and on rotten log (in Kunashir) ……………………………………………………………………………………… *F. conistipula*

-. Caducous leaves (both lobes and lobules) occuring in different parts of stems, rarely on flagelliform lateral branches. Well-developed leaf lobules with less unequal to subequal mouth valves. Styli filiform, subulate or triangular ………………………………………… 12

12. Leaf lobes often more or less squarrose when moist and dry; lobules often longer than wide, widest near or above middle; mouths often nodding towards stem, straight or truncate, usually with almost equal valves, though sometimes with the ventral side longer than the dorsal side. Androecia and perianths rarely present (in China often present) ……………………………………………………………………………................ *F. parvistipula*

-. Leaf lobes imbricate, proliferating marginal cells rare or absent. Lobules mostly parallel to the stem with considerably unequal mouth valves, in which the dorsal side is distinctly longer than the ventral side ….................................................................................. 13

13. Perianths trigonous, ornamentation smooth or mostly so. Leaf lobules mostly longer than wide …………………………………………………………………... *F. eboracensis*

-. Perianths with accessory keels and conspicuous tuberculae, or perianths unknown. Leaf lobules mostly subisodiametric ……………………………………………………….... 14

14. Perianths conspicuous armed …………...………………………………………….. 15

-. Perianths unknown …………………...………………………….…............................. 17

15. Perianths densely tuberculate and spinose (spines up to 6–9 cells long). Well-developed leaf lobules mostly subrotund, subquadrate to obovate, widest near or above middle. Styli subulate, 5 to 8 cells long and 3 to 4 (to 5) cells wide ........................... *F. tibetica*

-. Perianths densely tuberculate (tubercules 1–4 cells long) ………….......................... 16

16. Gynoecial bracts plane (rarely squarrose) when wet, mouth of perianth beak not or slightly flaring. Leaf lobules semicircular, mostly widest near or above the middle. Styli small, lanceolate-subulate, 2–3(–4) cells wide at base. ....................................... *F. appalachiana*

-. Gynoecial bracts strongly squarrose when wet. Mouth of perianth beak flaring. Well-developed leaf lobules mostly conical to somewhat hooked, widest near or below middle. Styli mostly filiform ………………………………………………………... *F. virginica*

17. Leaf lobes often with narrowly reflexed margins, especially near shoot apex (sometimes recognizable even when dry). Well-developed underleaves up to 1.33× wider than long, slightly narrowed toward base ………………………………........... *F. fukuzawana*

-. Leaf lobes mostly with flat margins. Underleaves usually longer than wide, rarely subisodiametric, cuneately narrowed toward base ……………………...…...….. *F. caucasica*

## 5. Taxonomy

### 5.1. Frullania parvistipula Steph., Sp. Hepat. 4: 397 (1910)

**Type citation**: Japonia [Japan], Provincia Tosa.

**Type specimens**: Japan [Shikoku], Nishi-harami, Tosa, 33°41′05″ N, 133°22′54″ E, 9 April 1905, *S. Okamura 170* (G00067235!, lectotype designated by Bonner [36] (p. 395).

**Illustrations**: Figure 2, Figure 3 and Figure 4C,D. Hattori [32], (Figure 212).

**Description**. Plants from dark copper-red-brown to dark-brown and almost black, partially scorched-like but sometimes with leaves in the upper parts of the shoots, light green at base, irregularly numerously 2–3 times branched, branches of *Frullania* and *Lejeunea* types, forming closely adhered to substratum mats, variable in size, usually small (in Russia, Taiwan, and the U.S.A. collections, as well as most collections from continental China), rarely (in *Mamontov 737-1-2*) rather large and well-developed, main shoots to 1.17 mm wide in sterile parts, up to 1.1 mm wide in gynoecial area (just below perianth), with branches ca. 0.4 mm wide. Stem 90–170 µm in cross-section, cells in the middle (10–)12–14 × 17–20 µm, outer cells (14–)16–20 × (20–)25–30 µm, cells on dorsal side thick-walled, subquadrate to elongated, ca. 18 × 18 µm to 17 × 25–30 µm. Leaf lobes wide spreading, remotely to contiguously inserted, in ventral view plane or convex, or concave, obliquely ovate to elliptical, 180–780 µm long, 124– to 120–700 µm wide, (1.0–)1.1–1.45× as long as wide, the apex rounded, the base crossing the stem, not or widely extending to the edge of stem, not auriculate at base. Median lobe cells almost isodiametric to slightly elongate, 13–14(–16) × 15–18(–20) µm, basal cells 15–16(–18) × 20–25(–30) µm, cell walls thin, straight to somewhat sinuous, with concave or convex trigones, often with intermediate thickenings; marginal cells almost isodiametric, somewhat smaller than the median ones, (10–)12–14 µm; margins often with rhizoid-like colorless unicellular hairs 7–10 µm wide and 20–50(–75) µm long. Cuticle smooth. Oil bodies 2–3(–4) per cell, spheroidal, 3–5 µm in diameter, or ellipsoidal and 3–4 × 6–7(–9) µm, at base of leaf 3–4(–5) per cell, slightly larger and 5–6 × 6–9 µm. Stem leaf lobules always inflated (helmet-shaped), explanated lobules not observed; the lobules slightly distant, with its apical part not overlying the stem, more or less parallel to stem or distinctly inclined at an angle up to 40 degrees (Figure 2, Figure 3 and Figure 4), very variable even on one plant from helmet-shaped to pitcher-shaped, usually longitudinally elongated, (75–)150–370 µm long, 100–350 µm wide, (0.75–)1.05–1.48× as long as wide, vault widely rounded or characteristically angled (Figure 2G,L and Figure 4C,D); the lobules widest in the upper half or near the middle in biggest lobules (Figure 2B,F,G,J,K and Figure 3B,F–H), or in lower third in smaller lobules (Figure 3G and Figure 4C,D); the mouth open, more or less flattened at the sides, straight or truncate; the mouth valves entire, almost equal, or dorsal valve slightly longer than the ventral one, almost not extending beyond it; the lobules sometimes constricted just above the mouth whose abaxial portion is obtusely protruding (Figure 2L,E, Figure 3G and Figure 4C,D). Cells of lobules (10–)12–15(–17) × 15–18(–20) µm, with sinuous walls and distinct intermediate thickenings. Styli triangular, 2–3 cells wide at the base, then a single cell wide of 4–5 superimposed subquadrate cells, ending with a mucilage papilla. Stem underleaves distant with one small blunt tooth on both sides, slightly narrowed to the base (on branches sometimes with parallel sides), 170–320 µm wide, 200–340 µm long, ca. 1.5–2(–2.5) times wider than stem, 0.9–1.14× as long as wide, divided up to 0.3–0.5 of their length by acute sinus into two obtuse to cuspidate lobes ending in extreme cases in 3–4 superposed almost isodiametric cells. Rhizoids colorless, long, and numerous (in Yunnan specimens) or rather scattered (in Guangxi specimens). Asexual reproduction via caducous leaves that fall off very easily and via gemmae-like structures of several cells (Figure 2A) which can grow directly on the leaves into small shoots (in *Mamontov 722-1-4*, numerous gemmae- and rhizoid-like colorless unicellular hairs are on almost all leaves, bracts, and bracteoles). Sexual condition dioicous. Androecia on abbreviated lateral branches, with one to several pairs of vegetative leaves, usually compact, ca. 0.6 mm wide and 0.7–1 mm long. Bracts in 3–6 pairs, ventricose with subequally obtuse lobes, with small one-celled teeth near the base, body of antheridium ca. 150 × 160 µm, antheridial stalks uniseriate, rather long (of ca. 30 cells). Female bracts in 2–3 pairs, dorsal lobe 360–600 µm wide, 500–800 µm long, with apices acute or obtuse or broadly rounded, lobules connate for 0.3–0.4 its length with lobe, narrow lanceolate, 500–580 µm long and from 150–220 µm at base to 50 µm near apex, with plane to distinctly revolute margins and large stylar tooth 4–6 cells at base and 10–12 cells long. Bracteole sometimes connate with one bract to 0.35 of the bracteole length, 230–420 µm wide, 440–560 µm long, divided to 0.27–0.43 of the length by acute or rounded sinus into two acute lobes; the bracteole margins usually with an acute tooth on one side (Figure 2C and Figure 3D,E). Perianth on leading stem, with one innovation some distance below, exserted for 0.5–0.7 its length beyond the bracts, 700–750 µm wide and 900–1100 µm long, four-keeled, the keels smooth, the lateral keels sharp, the ventral keels broad and two angled; the beak rather low, 25–50 µm high, the cells of the beak colorless, very soon destroyed. Capsule short-exserted, seta rather massive ca. 250 µm in diam. with ca. 32 rows of cortical cells, 8 cells in diam., elaters ca. 10–12 per valve (description of sporophyte based on *Mamontov 719-1-1*).

**Distribution**. According to the molecular results and study of herbarium specimens, *F. parvistipula* occurs in North America and East Asia. However, so far, we have a very incomplete understanding of its complete distribution in Asia, which necessitates a revision of *F. parvistipula*-like specimens from China, Mongolia, the Korean Peninsula, and Japan. In East Asia, the species is known in Japan (Shikoku, Kōchi Prefecture), from where it was described; besides Japan, it was recorded from the Korean Peninsula in Choi et al. [62]. Hattori & Lin [40] also recorded it from the Primorye Territory (sub ‘Regio Ussuriensis’) of Russia, the Guizhou, Hunan, Shandong, Xizang, and Yunnan Provinces of China, and also from Thailand and Bhutan. The records of the species from the Caucasus and Northern Italy in Hattori & Lin [40] most likely belong to *F. caucasica*. In our study, the species is confirmed in Asia for Taiwan (Chiayi and Nantou counties), continental China (Yunnan Province and Guanxi-Zhuang Autonomous Region), and Russia (Republic of Buryatia and Trans-Baikal Territory). The westernmost North Asian localities of *F. parvistipula* are those in the Selenga Highlands (107° E) and Ulan-Burgasy Range (108° E) in the Republic of Buryatia, Russia, whereas in East Asia, the species occurs up to 101° E in Xinping Yi and Dai Autonomous County in Yunnan. In North America, the species is confirmed for Arizona. It should be kept in mind, however, that this species is morphologically similar to other taxa with caducous leaves occurring in North America, namely *F. appalachiana*, *F. caucasica*, and *F. eboracensis*. Therefore, it is necessary to revise the collections of these species in North American herbaria and to sequence DNA from a larger number of samples across more regions. In North Asia, the range of *F. parvistipula* closely borders the range of *F. caucasica* in the Republic of Buryatia, Russia, so more research is needed to establish its precise distribution boundaries.

**Ecology**. In Yunnan and Guangxi, *F. parvistipula* occurs in mountain broadleaved and mixed broadleaved–coniferous forests at altitudes from 670 m a.s.l. in Guangxi to ca. 2300 m a.s.l. in Yunnan. It occurs there on tree trunks (including *Aleurites montana* (Lour.) Wils., *Castanopsis delavayi* Franch.), together with *Acrolejeunea* sp., *F. ericoides*, *F. neurota* Taylor, and *F.* cf. *rhystocolea* Herzog. In Taiwan, this species occurs on the bark of hardwood trees and *Liquidambar formosana* Hance in mixed forests and on secondary woody vegetation, at altitudes of ca. 1120–1390 m a.s.l. In Japan, the type specimen of *F. parvistipula* was collected on a tree, probably at low altitudes (according to the description of the locality in the G herbarium database). In Eastern Siberia and North America, *F. parvistipula* was collected only on rocks, i.e., in the Republic of Buryatia and the Trans-Baikal Territory, the species occurs in *Pinus sylvestris* L. and *Larix dahurica* Turcz. forests at altitudes from 495 to 1239 m a.s.l., often together with other liverworts: *Barbilophozia barbata* (Schmidel ex Schreb.) Loeske, *Cephaloziella konstantinovae* Mamontov & Vilnet, *Frullania austinii*, *F. cleistostoma* Schiffn. & W.Wollny, *F. conistipula*, *F.* ‘*muscicola’*, *Lophocolea minor* Nees, *Metzgeria pubescens* (Schrank) Raddi, *M. temperata* Kuwah., and *Porella platyphylla* (L.) Pfeiff. In Arizona, *F. parvistipula* has been found in pure mats in pine–oak forests at altitudes from 1680 to 1764 m a.s.l.

**Differentiation.** The specimens of *F. parvistipula* demonstrate the same *dilatata*-type morphology as numerous other Northern Holarctic species of subgenera *Chonanthelia*, *Frullania*, *Frullaniopsis*, and *Trachycolea* [11] occurring in the same or similar climatic conditions. Therefore, it can be difficult to distinguish *F. parvistipula* from several species, which have a similar morphology. In the specimens collected by the first author in Yunnan and Guangxi, no co-occuring species have been found that are habitually similar to *F. parvistipula.* However, *F. tibetica* was recently described as occurring in epiphytic habitats (like *F. parvistipula*) in the Xizang (Tibet) Autonomous Region of China. The distribution and ecology of *F. tibetica* are still poorly known as this species was described from a single collection from Bomê County in Xizang. Moreover, it is unknown whether *F. parvistipula* occurs in this region, so the joint occurrence of both species in Tibet cannot be excluded. Perianthous shoots of *F. tibetica* can be distinguished from *F. parvistipula* in the densely tuberculate and spinose perianth ornamentation (vs. smooth perianths in *F. parvistipula*). Sterile plants of *F. tibetica* differ from those of *F. parvistipula* in the (1) lobule position, which is parallel or slightly inclined toward the stem vs. inclined away from the stem in *F. parvistipula*, (2) the shape of the leaf lobule mouth, which is sometimes more or less constricted in comparison with the inflated upper half of the lobule [18] (p. 307, Figure 1G,J), and the dorsal valve of the mouth is usually slightly to distinctly longer than the ventral valve (in *F. parvistipula*, the mouth valves of the lobule mouth are subequal, and sometimes the ventral valve is slightly longer than dorsal), and (3) the styli, which are subulate, 5 to 8 cells long, 3 to 4(–5) cells wide vs. filiform to subulate, 4 to 5 cells long, 1 to 2(–3) cells wide in *F. parvistipula.* In the U.S.A., Japan, and Russia, there are also *F. caucasica*, *F. conistipula*, *F. fukuzawana*, and *F. koponenii*, which are distributed in the same or adjacent regions as *F. parvistipula*, and hence subject to confusion. In both Asia and North America, the ranges of *F. parvistipula* and *F. caucasica* have still not been found to overlap with one another, although the borders between these ranges are located rather close together. Indeed, in the USA, *F. parvistipula* was discovered in Arizona, whereas *F. caucasica* has been found in several localities in New Mexico and Colorado. In Siberia, the most easterly localities of *F. caucasica* are in the Khamar-Daban Range (ca. 105° E); thus, they are located rather nearby to the Selenga Highlands (ca. 107° E) and the Ulan-Burgasy Range (ca. 108° E), where the most westerly Siberian localities of *F. parvistipula* have been discovered. The proximity between the localities of both species in eastern Siberia and the USA. points to the possibility of finding both species in the same place. Moreover, in eastern Siberia, *F. conistipula* and *F. koponenii* occur in the same areas (including the same river valleys and even the same rock outcrops) as *F. parvistipula*, and in several specimens from the Republic of Buryatia (*Mamontov 398-1-9*, *415-1-8784*) and the Trans-Baikal Territory (*Mamontov 313-7-6643*), *F. conistipula* and *F. parvistipula* were found together. These circumstances necessitate the utilization of clear morphological distinctions between these four species for accurate determinations. Except for the well-developed shoots in the Yunnan specimen, *Mamontov 737-1-2*, all other studied specimens of *F. parvistipula,* particularly those from Japan, Arizona, and eastern Siberia, contain rather weak phases, which are relatively small and do not exceed 0.7 mm, instead being mostly 0.4–0.5 mm wide. In comparison, in the mentioned species of *F.* sect. *Trachycolea* (*F. caucasica*, *F. conistipula,* and *F. koponenii*) and in *F. fukuzawana*, the leafy shoots are usually 0.6–0.9 mm wide (but often wider). In our opinion, the leaf lobules of *F. parvistipula* demonstrate some basic morphological trends characteristic of species of *F*. sect. *Australes*, namely: (1) leaf lobules are usually longer than wide and (2) the mouth valves are usually equal in length, so the valves mostly do not extend beyond each other. These characteristics could help to distinguish sterile specimens of *F. parvistipula* from weak sterile phases of species of *F.* sect. *Trachycolea*, because in species of the latter section, the leaf lobules are often as long as wide, or wider than long, with mouth valves that are usually unequal, sometimes strongly so [33] (p. 163), [15] (pp. 133, 136, 138, 140, 152), [17] (p. 475). In the majority of the studied specimens of *F. parvistipula*, the leaf lobes are squarrose when wet, and the leaf lobes and lobules are strongly caducous in all parts of the shoots (Figure 2C,E,L and Figure 3A), but without bearing the dualistic morphology of the shoots as in *F. conistipula* (Figure 5D and Figure 6F,H) and *F. koponenii* [33] (p. 163, Figure 249: a–c), where the leaves are mostly persistent in the lower (in acropetal order) parts of shoots; in the upper parts of shoots and on some branches, almost all leaves have fallen off, with almost naked axes and only persistent underleaves. *Frullania koponenii* can be distinguished from *F. parvistipula* by the rather large, inflated, and long exserted perianth [33] (p. 163, Figure 249). *Frullania caucasica*, *F. conistipula*, *F. fukuzawana*, and *F. koponenii* differ from *F. parvistipula* in the rounded bract lobes (in *F. parvistipula*, the bract lobes are obtuse to acute at the apex). The sterile plants of *F. conistipula* and *F. koponenii* differ from those of *F. parvistipula* in the occurrence of their caducous leaves, mostly on specialized flagellae (somewhat similar to those in *F. austinii* and *F. bolanderi* Austin), which in *F. koponenii* are often erect or bent back to the shoot base. The Siberian specimens of *F. koponenii* from rocky habitats (and sometimes from the bark of trees) differ from *F. parvistipula* in the leaf lobules, which are mostly transversely elongated (that is, wider than long) and similar in shape to those of *F. austinii*. However, in the Russian Far East, only corticolous plants of *F. koponenii* have so far been found; these plants have leaf lobules that are usually longitudinally elongated (that is, longer than wide), like those in *F. parvistipula.* The Far Eastern plants of *F. koponenii* can hardly be confused with *F. parvistipula* since they usually have a much larger size, and usually bear long inflated perianths and characteristic flagellae.

Distinguishing between *F. parvistipula* and weak phases of *F. conistipula* is rather problematic in Siberia, since the latter sometimes bears longitudinally elongated leaf lobules and rather small styli. However, the leaf lobes in *F. conistipula* are not squarrose; moreover, in all the Siberian specimens containing weak phases of *F. conistipula*, shoots have been found with foliaceous styli (from one to several) and the leaf lobules have clearly to strongly unequal mouth valves, which are the characteristic features of *F. conistipula.* Weak phases of *F. appalachiana*, *F. eboracensis,* and *F. virginica* also sometimes have leaf lobules that are longer than wide, with subequal mouth valves. However, in specimens of the latter three species, the lobules of its well-developed shoots distinguish these species from *F. parvistipula.* Moreover, perianths are often found in specimens of all three species, of which only *F. eboracensis* can be confused with *F. parvistipula* due to its always smooth perianth surface. Furthermore, the former species is characterized and illustrated as having bract lobes that are rounded at the apices [15] (pp. 135, 137), while the bract lobes in the studied specimens of *F. parvistipula* typically have obtuse to acute apices.

**Specimens examined**. CHINA: Guangxi Zhuang Autonomous Region: Héchí District, 25°06′52.9″ N, 106°41′25.6″ E, 642 m a.s.l., 13 April 2019, *Mamontov 704-1-6401* (MHA). Bǎisè District, (1) 24°51′04.6″ N, 106°28′28.9″ E, 1112 m a.s.l., 14 April 2019, *Mamontov 710-1-5673* (MHA), *710-1-5675* (MHA), *710-1-5675* (MHA), *710-1-6479* (MHA), *710-1-6480* (MHA), *710-1-6481* (KPABG), *710-1-6482* (MHA), *710-1-6483* (MHA), *710-1-6484* (MHA); (2) 24°06′05″ N, 106°34′00.6″ E, 748 m a.s.l., 15 April 2019, *Mamontov 715-1-5674* (MHA), *715-1-5676* (MHA), *715-1-6474* (MHA); (3) National Park “Red Leaves Forest”, 23°21′43.4″ N, 106°38′59″ E, 669 m a.s.l., 16 April 2019, *Mamontov 716-1-1* (MHA); (4) 23°24′30″ N, 106°02′02.6″ E, 884 m a.s.l., 16 April 2019, *Mamontov 719-1-1* (MHA, KPABG), *719-1-6475* (MHA), *719-1-6476* (MHA), *719-1-6477* (MHA). Taiwan: Nantou County, Juji Twp., Jijidashan, summit region, 23°51.3′ N, 120°50.2′ E, 1390 m a.s.l., 18 October 2016, *A. Schäfer-Verwimp 37716* (MHA). Chiayi County, Alishan Range, Shihgupan River basin, along the first 1.2 km section of the Shihmenggu Trail, 23°34.1–2′ N, 120°46.0–4′ E, 1120–1250 m a.s.l., 22 October 2016, *Schäfer-Verwimp 37938* (MHA). Yunnan Province: Wenshan Zhuang and Miao Autonomous Prefecture, 23°43′40″ N, 104°54′27.7″ E, 1804 m a.s.l., 19 April 2019, *Mamontov 722-1-4* (MHA, KPABG), *722-1-6478* (KPABG). Xinping Yi and Dai Autonomous County, 24°05′38″ N, 101°54′34.7″ E, 1988 m a.s.l., 21 April 2019, *Mamontov 725-1-3* (MHA). Kunming District, Song Ming County, mountain near Guo Dong Shan Village, 25°23′56″ N, 102°43′09″ E, 2282 m a.s.l., 27 April 2019, *Mamontov 737-1-2* (MHA, KPABG), *737-1-6472* (KPABG), *737-1-6473* (MHA). Xishan District, Xi Shan Mt., 24°57′11″ N, 102°37′23″ E, 2500 m a.s.l., 21 March 1957, *V.I. Polyansky s.n.* (MHA). RUSSIA: Buryatia Republic: Mukhorshibirsky District, Selenga Highlands, Zaganskiy Range, Altacheiskiy Sanctuary, Altasha River valley, 50°59′38.8″ N, 107°12′20.7″ E, 635 m a.s.l., 5 August 2016, *Mamontov 662-2-2* (JE). Tarbagataysky District: (1) Selenga River valley, 51°32′17.0″ N, 107°20′51.2″ E, 564 m a.s.l., 25 July 2013, *Mamontov 374-2-8350* (MHA), *374-2-8768* (MHA), *374-2-8769* (MHA); (2) Ganzurinskiy Range, 51°50′51.7″ N, 107°10′48.8″ E, 654 m a.s.l., 25 June 2014, *Mamontov 420-1-8817* (MHA). Pribaikal′skiy District, Lake Baikal shore, near Turka Town, 52°56′51.9″ N, 108°13′24.2″ E, 473 m a.s.l., 26 June 2014, *Mamontov 423-1-9166* (MHA). Barguzinsky District, Lake Baikal shore, Gorevoi Cape, 53°15′24.0″ N, 108°32′09.7″ E, 462 m a.s.l., 28 June 2014, *Mamontov 426-1-9173* (MHA), *423-1-9174* (MHA). Khaimskiy District, Ulan-Burgasy Range, 52°38′54.2″ N, 108°03′17.2″ E, 495 m a.s.l., 26 July 2013, *Mamontov 376-1-1* (MHA), *376-1-7352* (MHA), *376-1-7353* (MHA). Khorinsk District: (1) Kurbinskiy Range, Khorinsk Town surroundings, 52°08′53.3″ N, 109°43′27.6″ E. 659 m a.s.l., 23 June 2014, *Mamontov 414-3-8778* (MHA), *414-3-8779* (MHA), *414-3-8780* (MHA); (2) Kurbinskiy Range, Kurba River valley, Thegda Town surroundings, 52°24′49.2″ N, 108°54′22.9″ E, 646 m a.s.l., 23 June 2014, *Mamontov 415-1-8784* (MHA), *415-2-8787* (MHA), *415-2-8788* (MHA), *415-2-8792* (MHA), *415-2-8793* (MHA), *415-2-8795* (MHA), *415-2-8796* (MHA), *415-2-8797* (MHA), *415-2-8798* (MHA); ibid., 52°28′25.9″ N, 108°50′03.7″ E, 839 m a.s.l., 24 June 2014, *Mamontov 416-2-7133* (MHA). Kurumkansky District, Dzherginskiy State Reserve, Southern Muya Range, Dzhirga River basin, 54°54′46.4″ N, 111°14′24.6″ E, 629 m a.s.l., 3 August 2013, *Mamontov 395-1-2* (KPABG), *395-1-6* (KPABG), *395-2-2* (MHA), *395-3-1* (MHA), *395-3-4* (MHA), *395-3-5* (MHA); ibid., 54°54′50.5″ N, 111°19′51.4″ E, 598 m a.s.l., 8 August 2013, *Mamontov 397-1-26* (MHA); ibid., 54°54′59.3″ N, 111°12′32.4″ E, 632 m a.s.l., 9 August 2013, *Mamontov 398-1-6* (MHA), *398-1-9* (MHA). Trans-Baikal Territory: Uljetovsky District, Ingoda River valley, 15 km S of Tanga Settlement, 50°52′ N, 111°34″ E, 842 m a.s.l., 12 July 2007, *Afonina 04607-3* (KPABG). Agin-Buryat Okrug, National Park “Alhanai”, Daurskiy Range, Arschan Creek, 50°51′ N, 113°23′ E, 1171 m a.s.l., 23 July 2007, *Afonina 08107-1* (KPABG). Kalarsky District, Stanovoy Highlands, Srednij Sakukan River valley, Chara Town vicinities, 56°52′32.4″ N, 118°12′10.2″ E, 736 m a.s.l., 14 August 2012, *Mamontov 313-7-6643* (MHA). Karymskiy District, Daurskiy Range, Aratsagon Mt., 51°54′51.2″ N, 114°25′05.5″ E, 892 m a.s.l., 13 July 2012, *Mamontov 262-6-6100* (MHA), *262-7-6091* (MHA), *262-12* (MHA). Kyrinsky District, Sokhondinskiy State Reserve, Khentey-Chikoyskoye Nagor’e Uplands, (1) Ende River valley, lower of Khukhje-Bajtsa Brook, 49°27′50.2″ N, 110°52′11.2″ E, 1214 m a.s.l., 27 August 2011, *Mamontov 182-1-1* (MHA), *182-6-1* (KPABG-118618), *182-7-6838* (MHA), *182-10-4* (MHA), *182-12-6794* (MHA), *182-13-6821* (MHA), *182-13-6844* (MHA), *182-14-1* (KPABG), *182-16-1* (MHA); ibid., 49°26′39″ N, 110°56′55.3″ E, 1162 m a.s.l., 27 August 2011, *Mamontov 184-61-1* (MHA), *186-2-7155* (MHA), *186-2-8905* (MHA); (2) Shanarichi Creek, 49°25′57.5″ N, 110°52′55.5″ E, 1239 m a.s.l., 29 August 2011, *Mamontov 187-18-1* (LE); (3) Ende River valley, 49°27′ N, 110°51′ E, 1320 m a.s.l., 12 July 2010, *Afonina 1910-5* (KPABG); (4) Agutsa River valley, 49°40 ′ N, 111°26′ E, 1098 m a.s.l., 18 July 2010, *Afonina 3710-1* (KPABG, LE), *3710-2* (KPABG, LE), *3710-3* (LE), *3710-5* (LE); ibid., 49°40′ N, 111°26′ E, 1300 m a.s.l., 23 July 2010, *Afonina 6010-10* (KPABG), *6010-18* (KPABG), *6010-21* (KPABG). U.S.A.: Arizona: Cochise Co., USDA Forest Service, Coronado National Forest, Chiricahua Mountains, entrance to “Log” Canyon, 31°51′10″ N, 109°11′35.1″ W, 1680 m a.s.l., 29 March 2007, *J. Brinda 1396b* (MHA, MO), *1398* (MHA, MO). Santa Cruz Co., USDA Forest Service, Coronado National Forest, Mount Wrightson Wilderness, upper Madera Canyon, 31°42′29.5″ N, 110°52′10.1″ W, 1764 m a.s.l., 9 April 2017, *Brinda 9978* (MHA, MO).

### 5.2. Frullania conistipula Steph., Sp. Hepat. 4: 399 (1910)

**Type citation**: Japonia [Japan], Provincia Aomori.

**Type specimens**: Japan, Aomori, Tohoku, 38°03′24″ N, 139°50′35″ E, 20 May 1898, *U.J. Faurie 135* (G00069157!, lectotype designated by Bonner [36] (p. 271).

**Illustrations**: Figure 4A,B,E–J, Figure 5 and Figure 6.

**Description**. Plants yellow-green or reddish brown and somewhat glossy, becoming brown in the oldest portions, slender, main shoots prostrate, up to 20 mm long, with sterile shoots 0.7–0.9 mm wide and fertile shoots below the gynoecia up to 1.3 mm wide, stems irregularly 1–2-pinnate, branches evenly spaced, short branches usually denuded and wiry, 1.1–1.7 mm long, often ascending at their tips, foliate branches spreading at oblique angles, longer and often branched again, with their tips often denuded. First branch appendage consists of a bifid, asymmetric, ovate-lanceolate segment. Second branch appendage lateral and consisting of two galeate segments or a galeate segment and a flat, short-lanceolate segment. Stems rounded, 120–165 µm in diameter, yellow, dorsal epidermis 5–7 cells wide, consisting of firm-walled, mostly rectangular cells, 30–43 × 16–18 µm, ventral merophyte approximately 6 cells wide; stems in cross-section approximately 8–9 cells wide, cells oblong to subquadrate, 15–25 × 12–25 µm, cortical cells somewhat colored and collenchymatous with yellowish walls, medullary cells hyaline with pale walls. Leaves imbricate or touching when attached, sometimes becoming remote on the oldest.

Shoot portions, frequently caducous on short branches, sometimes or often caducous in the upper stem and upper portions of long branches. Dorsal lobes broadly ovate to reniform, 450–580 × 600–830 µm, slightly concave, the apices broadly rounded and slightly decurved, margins entire, antical base auriculate, extending beyond the distal edge of the stem by approximately the stem width. Marginal cells subquadrate, rarely oblong, 15–18 × 11–15 µm, sometimes bulging and pigmented, and then 20–25 × 15–17 µm, median cells regularly polygonal, 17–30 × 11–20 µm, with moderately to distinct bulging trigones and mostly distinct intermediate thickenings, basal cells sometimes colored and more elongated, 28–42 × 18–24 µm, with distinct trigones; cuticle smooth; oil bodies granular, 3–4(–6) per cell, spherical, and approximately 5 µm, or irregularly ellipsoidal and 7–8 µm; ocelli lacking. Lobules inserted parallel to the stem or slightly inclined toward it, consistently inflated, galeate, 270–330 × 210–280 µm, about as long as wide or wider than long and then widest in lower third, sometimes longer than wide, and then widest near the middle, apex conical, compressed above the mouth, sometimes strongly so and forming a non-protruding, blunt beak, mouth valves unequal, often significantly separated with the dorsal side considerably longer than the ventral side. Styli foliaceous, 10–14(–20) cells long and 3–5(–9) cells wide, sometimes triangular, with (2)-3–6 cells long. Underleaves broadly obcuneate, 230–340 × 180–310 µm, approximately 1.5 times as wide as the stem, bifid for 50–65 µm, sinus broadly acute or U-shaped, lobes triangular, acute, lateral margins plane, entire or with a sharp angulation or tooth on both sides above the middle of the leaf. Rhizoids sparse, hyaline to yellowish, in fascicles from the base of the underleaves. Specialized asexual reproduction by caducous leaves, detached lobes often with thick-walled, short, hyaline rhizoids (50–75 µm long), arising from bulging marginal cells. Sexual condition presumably dioicous (androecia not seen). Gynoecia terminal on the stem or main branch, immature bracts in 2–3 pairs, lacking subfloral innovation, unequally bifid and divided to about 0.6–0.8 their length, innermost bract lobe ovate, 590–630 × 355–460 µm, slightly concave, apex broadly rounded, margins plane and entire, lobule lanceolate, 410 × 230–310 µm, apex obtuse, margins entire except for a short but prominent stylar tooth near the base of the free margin, and sometimes a few short teeth or cilia; immature bracteole free from the bract or shallowly connate with the bract on one side, elliptical, 310 × 220 µm, bifid to about 0.3 the length, sinus acute, lobes triangular-lanceolate, somewhat acuminate, lateral margins plane, subentire or with distinct angulation on both sides. Perianth and capsule unknown.

**Distribution**. Aside from the type locality in northern Honshu, Japan, *F. conistipula* has been found in Siberia (Republics of Altai and Buryatia, and Trans-Baikal Territory, Russia) and in the southern regions of the Russian Far East, i.e., Primorye Territory and Sakhalin Region.

**Ecology**. In the Republic of Altai and in the south of Eastern Siberia (Republic of Buryatia and Trans-Baikal Territory, Russia), *F. conistipula* has been found on both shaded and exposed cliffs in larch (*Larix dahurica* Turcz.), pine (*Pinus sylvestris* L., *P. sibirica* Du Tour), and mixed (*Betula platyphylla* Sukaczev-*Picea obovata* Ledeb.) forests. Only once was the species collected in the soil at the base of a tree trunk in a mixed forest in the southern part of the Trans-Baikal Territory (*Afonina A5710-1*). In Siberia, the species was found in pure mats or in association with numerous other liverworts, most often *Barbilophozia barbata* (Schmidel ex Schreb.) Loesk, *Frullania austinii*, *F. cleistostoma*, *F. davurica* Hampe ex Gottsche, Lindenb. & Nees, *F.* ‘*muscicola*’, *F. parvistipula*, *Cephaloziella konstantinovae*, *C. varians* (Gottsche) Steph., *Metzgeria pubescens* (Schrank) Raddi, rarely *Frullania koponenii*, *Lophoziopsis excisa* (Dicks.) Konstant. & Vilnet, *Metzgeria furcata* (L.) Dumort., *Porella platyphylla* (L.) Pfeiff., *Radula complanata* (L.) Dumort., *Reboulia hemisphaerica* (L.) Raddi, *Sphenolobus minutus* (Schreb. ex D. Crantz) Berggr., *Tritomaria exsecta* (Schmidel) Loeske, and *Trilophozia quinquedentata* (Huds.) Bakalin. In the Primorye Territory (the Russian Far East), *F. conistipula* has been found only on cliffs, both shaded and exposed, in larch (*L. cajanderi* Mayr), pine (*Pinus koraiensis* Siebold & Zucc.), oak (*Quercus mongolica* Fisch. ex Ledeb.), mixed (coniferous-broadleaved), and polydominant floodplain forests, and also on coastal cliffs shaded by alder thickets. The co-occurring species were *F. fukuzawana*, *F. jackii* Gottsche, *F.* ‘*muscicola*’, and *Porella ulophylla* (Steph.) S.Hatt. In Kunashir, this species was found only in pure mats on decaying wood in *Abies-Picea* forests with an admixture of *Betula ermanii* Cham.

**Differentiation**. Weak xeromorphous phases of *F. conistipula* are similar to *F. parvistipula*. However, there are significant differences in the lobule shape of these species. In *F. parvistipula*, the leaf lobules are often inclined from the stem (Figure 2F,J,L and Figure 4C,D), usually distinctly longer than wide and often widest near the middle or in the upper third and narrowed to the mouth, with straight to truncate mouths, usually with almost equal valves, of which the ventral one sometimes appears to be a little longer than the dorsal one, so the dorsal lobule margin is hidden under the ventral margin when viewing from the ventral side (Figure 2C,E, Figure 3A,G and Figure 4C,D). In *F. conistipula*, the weakly developed leaf lobules can be similar to those in *F. parvistipula* in their general habit (Figure 5E and Figure 6H), i.e., the lobules can be longer than wide and are sometimes widest near the middle. However, almost always, the lobules in *F. conistipula* are inserted parallel to the stem or inclined toward it and have unequal (often strongly so) mouth valves, of which the dorsal one is always longer than the ventral one. The well-developed leaf lobules in *F. conistipula* (Figure 4A,B,E,G–J, Figure 5A–D,F–I,K and Figure 6A–C,E–G,I,J) are usually conical, with lobules as long as wide or wider than long and widest in the lower third, with characteristically unequal mouth valves. The styli also differ in both of the compared species. In *F. parvistipula*, the styli are usually small and triangular to filiform, ca. 4–5 cells long, whereas in well-developed plants of *F. conistipula*, the styli are foliaceous, from 10 to 20 cells long. *Frullania caucasica* and *F. koponenii* are also morphologically similar to *F. conistipula*; however, the majority of specimens of *F. koponenii* are found on trees and usually have perianths. By comparison, *F. caucasica* and *F. conistipula* in Siberia have been found on rocks only, and the fertilized gynoecia and mature perianths are unknown for both species. Two of the sequenced specimens of *F. koponenii* (*Mamontov 511-1-2* and *Afonina A07208*) from the Trans-Baikal Territory were collected on cliffs on riverbanks, growing together with *F. davurica*. Moreover, a number of specimens determined as *F. koponenii* were collected on rocks in the Republic of Buryatia, including on the shore of Lake Baikal and in the East Sayan Mts., where the species was also found growing together with *F. davurica* [63]. The saxicolous specimens of *F. koponenii* contained no perianths, so their differentiation from *F. conistipula* (as well as from the saxicolous phases of *F. caucasica* and *F. fukuzawana*) is more problematic. Well-developed shoots of *F. conistipula* from shaded rock outcrops can be distinguished by the presence of rather large, foliaceous styli of up to 20 cells long and 9 cells wide (Figure 4, Figure 5 and Figure 6). In its northernmost locality, the studied plants of *F. conistipula* (*Mamontov 544-1-6073*, *544-1-7762*, *544-1-8420*) have only triangular styli, as well as leaf lobules that are somewhat wider than long and are exceptionally similar to those of *F. koponenii*, as illustrated by Hattori [33] (p. 163, Figure 249a). However, the presence of caducous leaves (or only caducous leaf lobules) scattered on the main shoot and branches apart from specialized flagellae allows for the determination of these specimens as *F. conistipula* [the similarly leaved shoot of *F. conistipula* from the sequenced specimen *Bakalin K-37-5-06* is shown in the Figure 6H]. This determination was later confirmed with molecular data (Figure 1). Further discussion on differences between *F. caucasica*, *F. fukuzawana*, *F. koponenii*, and weak phases of *F. conistipula*, with rather small triangular styli, is expected in a separate future study.

**Specimens examined**. RUSSIA: Altai Republic: Ulagansky District, Kurkure Range, Kayakkatuyarykskiy Creek basin, 51°05′ N, 88°11′ E, 2200 m a.s.l., 4 July 1991, *M.S. Ignatov 7/98* (MHA). Buryatiya Republic: Barguzinsky District, Lake Baikal shore, Gorevoi Cape, 53°15′24.0″ N, 108°32′09.7″ E, 462 m a.s.l., 28 June 2014, *Mamontov 426-1-7325* (MHA), *426-1-7326* (MHA), *426-1-7327* (MHA), *426-1-7339* (MHA), *426-1-7340* (MHA), *426-1-7341* (MHA), *426-1-9172* (MHA), *426-1-9242* (KPABG), *426-1-9245* (KPABG), *426-1-9246* (KPABG), *426-1-9247* (KPABG). Ivolginsk District, Khamar-Daban Range, Khalyuta River valley, 51°50′51.7″ N, 107°10′48.8″ E, 654 m a.s.l., 25 June 2014, *Mamontov 419-1-8811* (MHA), *419-1-8813* (KPABG), *419-2-7355* (MHA), *419-2-8815* (KPABG). Khaimskiy District, Ulan-Burgasy Range, 52°38′54.2″ N, 108°03′17.2″ E, 495 m a.s.l., 26 July 2013, *Mamontov 376-1-7353* (MHA), *376-1-8774* (MHA). Khorinskiy District, (1) Kurbinskiy Range, Kurba River valley, 52°24′49.2″ N, 108°54′22.9″ E, 646 m a.s.l., 23 June 2014, *Mamontov 415-1-8784* (MHA), *415-2-7338* (MHA); (2) Ulan-Burgasy Range, upper of Khail River, Thegda Town surroundings, 52°28′25.9″ N, 108°50′03.7″ E, 839 m a.s.l., 24 June 2014, *Mamontov 416-2-8801* (MHA). Kurumkansky District, (1) Dzherginskiy State Reserve, Southern Muya Range, Dzhirga River basin, 54°54′59.3″ N, 111°12′32.4″ E, 632 m a.s.l., 09.08.2013, *Mamontov 398-1-9* (MHA); (2) Barguzin Range, Ulyugna River valley, 54°57′43.1″ N, 111°00′08.7″ E, 773 m a.s.l., 26 June 2016, *Mamontov 570-1-5111* (KPABG), *570-1-5112* (MHA). Kyakhtinsky District, Selenga River valley, Naushki Town surroundings, 50°23′33.1″ N, 106°07′14.1″ E, 721 m a.s.l., 23 July 2013, *D.Ya. Tubanova Kяx-01/13-6179* (MHA). Selenginsky District, Khamar-Daban Range, Gusinoe Lake shore, 51°13′7.5″ N, 106°17′42.5″ E, 684 m a.s.l., 21 July 2013, *Tubanova Ce-03/13* (MHA). Severo-Baikalsky District, Barguzin State Reserve, Barguzin Range, (1) Davshe River basin, 54°23′45.7″ N, 109°30′24.6″ E, 984 m a.s.l., 17 July 2014, *Yu. S. Mamontov 450-1-5517* (MHA), *450-1-5521* (MO), *450-1-5592* (MHA), *450-1-5648* (KPABG), *450-1-5649* (KPABG), *450-3-1* (MHA); (2) Shumilikha River valley, 54°04′33.9″ N, 109°37′11.1″ E, 1500 m a.s.l., 13 July 2014, *Mamontov 438-1-11* (MHA). Trans-Baikal Territory: Agin-Buryat Area, National Park “Alhanai”, Alhanai Mt., 50°50′ N, 113°24′ E, 11 July 2006, *O.M. Afonina A3506-1* (LE), *A3506* (KPABG-112650). Kalarsky District, (1) Kodar Range, National Park “Kodar”, Syul′ban River valley, 56°38′0.4″ N, 117°11′50.8″ E, 1060 m a.s.l., 23 June 2015, *Mamontov 544-1-6073* (MHA), *544-1-7762* (MHA), *544-1-8420* (KPABG); (2) National Park “Kodar”, Srednij Sakukan River valley, Chara Town vicinities, 56°52′32.4″ N, 118°12′10.2″ E, 736 m a.s.l., 14.08.2012, *Mamontov 313-7-6643* (MHA); (3) Southern Muya Range, Koyra River valley, 56°13′54.6″ N, 115°52′19.6″ E, 574 m a.s.l., 5 August 2012, *Mamontov 309-20-7404* (MHA), *309-20-7405* (KPABG), *309-20-7406* (MHA). Krasnochikoysky District, (1) Chikoy National Park, Atsinskiy Range, Yugal River valley, 50°15′55.6″ N, 109°05′29.8″ E, 953 m a.s.l., 12 August 2011, *Mamontov 100-1-13* (KPABG), *100-2-1* (KPABG-116352). (2) Malkhanskiy Range, Cheremkhovo Pass, 50°44′04.7″ N, 110°22′19.1″ E, 1360 m a.s.l., 14 August 2011, *Mamontov 105-2-7* (KPABG), *107-1-3* (KPABG-118630). Kyrinsky District, Sokhondinskiy State Reserve, Khentey-Chikoyskoye Nagor’e Uplands: (1) Agutsa River valley, 49°40′ N, 111°26′ E, 1399 m a.s.l., 22 July 2010, *O.M. Afonina 5510-1* (KPABG-116339), *5510-2* (KPABG), *5510-6* (KPABG), *5510-8* (KPABG-118647); ibid., *Afonina 5710-1* (KPABG-116341); (2) Upper of Bukukun River, 49°37′ N, 111°00″ E, 1463 m a.s.l., 24 July 2007, *Afonina A04808* (KPABG-114162), *A04808-10* (KPABG-113909, under *F. austinii*), *A04808-2* (LE, sub *F. parvistipula*); *A04808-7119* (MHA); (3) Sokhondo River valley, 49°30′32″ N, 111°04′37.6″ E, 1205 m a.s.l., 23 August 2011, *Mamontov 166-2* (LE), *166-9* (MHA); (4) Ende River valley, 49°27′ N, 110°50′ E, 1161 m a.s.l., 15 July 2010, *Afonina A2910-3* (KPABG), *A2910-15* (KPABG), *A2910-22* (KPABG), *A2910-23* (KPABG), *A2910-26* (KPABG); ibid., *Afonina A3010-6* (KPABG-119778), *A3010* (KPABG-115249); (5) Ende River valley, lower of Khukhe-Bajtsa Brook, 49°26′39″ N, 110°56′55.3″ E, 1162 m a.s.l., 27 August 2011, *Mamontov 183-3-8903* (KPABG), *183-8-7146* (MHA), *183-8-7147* (MHA), *183-9-7152* (KPABG), *183-12-6856* (MHA), *183-12-6857* (KPABG), *183-12-7330* (MHA), *183-12-7331* (MHA), *183-12-7332* (MHA), *183-12-7333* (MHA), *183-12-7334* (MHA); (6) Ende River valley, Shanarichi Creek, 49°25′57.5″ N, 110°52′55.5″ E, 1239 m a.s.l., 29 August 2011, *Mamontov 187-14-7160* (MHA), *187-8-6784* (MHA); (7) Tyrin River valley, Khapcheranga Town surroundings, 49°45′36.7″ N, 112°20′51.9″ E, 1057 m a.s.l., 29 August 2011, *Mamontov 189-13-8906* (MHA). Primorye Territory: Khasanskiy District, Krabbe Peninsula, 42°36′34.2″ N, 130°55′45.9″ E, 35 m a.s.l., 23 October 2020, *Mamontov 854-1-7314* (MHA). Olginsky District, Vatovskogo Peninsula, 43°52′59.2″ N, 135°30′39.1″ E, 8 m a.s.l., 14 August 2018, *Mamontov 697-1-4584* (MHA), *697-1-5406* (MHA), *697-1-5407* (KPABG), *697-1-5408* (MO), *697-1-5409* (MHA), *697-1-5411* (MO); ibid., 43°53′1.5″ N, 135°30′41.2″ E, 46 m a.s.l., 14 August 2018, *Mamontov 699-1-4622* (MHA). Terneysky District, Sikhote-Alin′ Nature Reserve, Sikhote-Alin′ Mts: (1) Yasnaya River valley, Maisa Cordon surroundings, 45°14′7.4″ N, 136°30′39.6″ E, 139 m a.s.l., 2 October 2019, *Mamontov 801-1-9* (MO), *801-1-5669* (MHA); ibid., 45°16′10.2″ N, 136°29′57.2″ E, 273 m a.s.l., 3 October 2019, *Mamontov 802-1-5665* (MHA); (2) Zabolochennaya River valley, 45°16′19.2″ N, 136°29′45.8″ E, 282 m a.s.l., 3 October 2019, *Mamontov 803-1-2* (MHA); ibid., 45°15′50.7″ N, 136°30′17.3″ E, 182 m a.s.l., 3 October 2019, *Mamontov 804-2-5533* (KPABG), *804-2-5666* (MHA), (3) Upolnomochennyi Cordon surroundings, 45°09′24.3″ N, 136°46′43.5″ E, 17 m a.s.l., 5 October 2019, *Mamontov 805-4-1* (MHA); ibid. 45°09′47.5″ N, 136°47′0.3″ E, 22 m a.s.l., 6 October 2019, *Mamontov 806-1-1* (MHA), *806-1-5534* (KPABG), *806-1-5537* (KPABG), *806-1-5668* (MHA); (4) Terney Town, 45°01′58.1″ N, 136°38′44.3″ E, 12 m a.s.l., 8 October 2019, *Mamontov 807-1-2* (MO), *807-1-3* (KPABG, sub *F. koponenii*), *807-1-4* (MHA), *807-1-7* (MHA), *807-1-4522* (MO), *807-1-5538* (KPABG), *807-1-5667* (KPABG), *807-1-5670* (MHA); ibid., 11 October 2019, *M.A. Kolesnikova 6897* (MHA). Sakhalin Region: Kunashir Island, Yuzhno-Kurilsk District, Kuril′skiy Nature Reserve, Dal′nij Stream, 44°27′41″ N, 146°06′49″ E, 100 m a.s.l., 28 August 2006, *V.A. Bakalin K-37-5-06* (KPABG, VBGI).

### 5.3. Frullania fukuzawana Steph. ex Mamontov, J.J.Atwood & Vilnet, sp. nov.

**Type specimen**: Japan, Chubu, Fukuzawa, Awa, 34°39′33″ N, 137°17′36″ E, 2 February 1913, *S. Okamura 422* (holotype: G00066779!). 

**Illustrations**: Figure 7.

**Diagnosis**: *Frullania fukuzawana* differs from *F. conistipula* in its shoots, with less frequent caducous leaves and a less dilated antical base of the dorsal lobe, as well as having shorter, subulate styli.

**Description**: Plants yellowish to brownish, irregularly branched (up to 2 times), with *Frullania* type branches, rather small, main shoots 600–840 µm wide in sterile parts, up to 1.27 mm wide in gynoecial area, with leafy branches 280–620 µm wide; stem 90–140 µm wide. Stem leaf lobes remotely to contiguously inserted, in ventral view plane or concave, obliquely ovate to elliptical, 255–454 µm wide, 390–520 µm long, ca. 1.13–1.52 times longer than wide, apex rounded, the base crossing the stem, slightly to widely extending beyond the edge of stem (Figure 7A,B), not auriculate at base. Median lobe cells almost isodiametric to slightly elongate, 17–21 × 24–29 µm, basal cells 19–21 × 21–31 µm, cell walls thin, straight to somewhat sinuous, with concave or convex trigones, with intermediate thickenings; marginal cells almost isodiametric, somewhat smaller than the median ones, 12–14 µm. Cuticle smooth. Stem leaf lobules all inflated (helmet-shaped), explanated lobules not observed; the lobules slightly distant, with its apical part not overlying the stem, more or less parallel to the stem or slightly inclined from it, 166–238 µm long and 166–220 µm wide, usually subisodiametric, (0.91–)1.05–1.11× as long as wide, widest in lower third, rarely in upper half; vault widely rounded; distinct beak absent; the mouth open, more or less flattened at the sides, straight or concave or convex; the mouth valves entire, the dorsal valve usually longer than the ventral one, extending beyond it; the lobules not or slightly constricted above the mouth. Styli triangular, 2–3 cells wide at the base, then a single cell wide of 4–5 superimposed subquadrate cells. Stem underleaves distant, armed on one or both sides with one or two acute or blunt teeth or angulations, slightly narrowed to the base, 200–340 µm long, 170–320 µm wide, ca. 1.5–2(–2.5)× wider than stem, 0.9–1.14× as long as wide, divided up to 0.3–0.5 of their length by acute sinus into two obtuse to acute (ending with one-celled tips) lobes. Rhizoids colorless. Asexual reproduction via caducous leaves. Probably dioicous (male plants not found). Female bracts in 2 pairs, dorsal lobe of the innermost bracts 500–810 µm long, 330–560 µm wide, with rounded apices, lobules connate for 0.28–0.43 its length with lobe, lanceolate, 360–680 µm long, 155–296 µm wide, plane to distinctly concave, with somewhat revolute margins and rather small stylus. The innermost bracteole sometimes connate with one bract to 0.22 of the bracteole length, 440–480 µm µm long, 135–200 µm wide, divided to 0.37–0.39 of the length by acute or obtuse sinus into two lobes; the lobes acute or obtuse at apex; the bracteole margins entire or with a rounded angulation on one side. Otherwise unknown.

**Ecology**. In Honshu and Kunashir, *F. fukuzawana* was collected on poplar trees near the seacoast (in Kunashir) or at ca. 1.5–2 km from the seacoast (in Honshu). In the Primorye Territory, Russia, the species was collected only on cliffs, but also mostly on the seacoasts, or on rock massif at ca. 1.5 km from the seacoast as an exception. In these places, the species was found mostly in pure mats, rarely together with other liverworts. On the coastal cliffs, the associates were *F. austinii* and *F. conistipula*, while the associates in the rock massif away from the seacoast were *F.* ‘*muscicola*’, *F. sinensis* Steph., *F. taradakensis* Steph., *Lejeunea japonica* Mitt., and *Syzygiella autumnalis* (DC.) K.Feldberg, Váňa, Hentschel & Heinrichs.

**Distribution**. The species is currently known from the southern parts of Honshu, Japan and the Russian Far East. However, rather wide occurrence of this species in the coastal part of the southern half of the Primorye Territory, Russia suggest it also likely occurs at least on the seacoasts of the Korean Peninsula, as well as in Japan, at least in Hokkaido and in northern part of Honshu.

**Comment**. The illustrations of this species based on the sequenced specimens and the description of its differences from similar species are expected in a separate future paper devoted to the discussion of the morphology of *F. caucasica*, *F. koponenii*, and *F. fukuzawana*.

**Specimens examined**. JAPAN: [Honshu] Tottori-ken, Tohaku-gun. Yurihama-cho. Urushibara, 35°28′55.1″ N, 133°56′9.3″ E, 42 m a.s.l., 11 March 2013, *Bakalin J-3-4-13* (VBGI, KPABG). RUSSIA: Primorye Territory: Khasanskiy District, (1) Zuby Drakona Rock Massif, 42°53′21.1″ N, 131°17′58.9″ E, 210 m a.s.l., 21 October 2020, Mamontov *850-1-6008* (MHA), *850-1-6009* (MHA), *850-1-6012* (MHA), *850-1-6020* (MHA), *850-1-6021* (MHA, KPABG-126089), *850-1-6022* (MHA), *850-1-6023* (MHA), *850-1-6024* (MHA), *850-1-6301* (MHA, KPABG-126112), *850-1-7290* (KPABG), *850-1-7291* (KPABG), *850-1-9117* (MHA), *850-1-9120* (MHA), *850-1-9121* (MHA, under *Lejeunea japonica*), *850-1-9122* (KPABG), *850-1-9123* (MHA); (2) Slavyanka Village surroundings, Bruce Cape, 42°52′41.6″ N, 131°28′8.1″ E, 8 m a.s.l., 22 October 2020, *Mamontov 851-1-6016* (MHA, KPABG-126113), *851-1-6017* (MHA), *851-1-9130* (MHA), *851-1-9131* (MHA), *851-1-9132* (KPABG), *851-1-9133* (KPABG); ibid., *Mamontov 852-1-6302* (MHA); (3) Reyd Pallada Bay, Khasan Settlement, near Mramornyi Cape, 42°34′23.2″ N, 130°47′11.3″ E, 17 m a.s.l., 22 October 2020, *Mamontov 855-1-6303* (MHA, KPABG-126088), *855-1-9124* (MHA), *855-1-9125* (MHA), *855-1-9126* (MHA), *855-1-9127* (KPABG), *855-1-9128* (KPABG), *855-1-9129* (MHA). Olginsky District, Vatovskogo Peninsula, 43°53′1.5″ N, 135°30′41.2″ E, 46 m a.s.l., 14 August 2018, *Mamontov 699-1* (MHA). Terneysky District, Sikhote-Alin′ State Reserve, Sikhote-Alin′ Mts., Upolnomochennyi Cordon, 45°09′47.5″ N, 136°47′0.3″ E, 22 m a.s.l., 6 October 2019, *Mamontov 806-1-1* (MHA, under *F. conistipula*), *806-1-2* (MHA), *806-1-5668* (MHA, under *F. conistipula*). Sakhalin Region, Kunashir Island, Yuzhno-Kurilsky District, Kuril′skiy State Reserve, Tret′jakova River valley, 43°59′0.5″ N, 145°38′50.1″ E, 14 m a.s.l., 21 September 2020, *Mamontov 815-1-5627* (MHA), *815-1-5628* (MHA, KPABG-126092), *815-1-5629* (MO), *815-1-6018* (MHA), *815-1-6019* (KPABG).

### 5.4. Frullania aberrans (C. Massal.) Mamontov, Vilnet & J. J. Atwood, comb. et stat. nov.

**Basionym**: *Frullania aeolotis* var. *aberrans* C. Massal., Mem. Accad. Verona 73(2): 39 (1897).

**Type citation**: China “in montibus Lun-san-huo et Si-ku-tzui-san, nec non prope Sce-kin-tsuen in prov. Schen-si, Chinae interioris leg. Rev. Pater Giraldi”. 

**Type Specimens:** China interior, provincia Schen-si [Shaanxi] sept., in summo monte Lun-san-huo, November 1875, *J. Giraldi 29* (BM– lectotype designated by Hattori & Lin [40] (p. 141); G00264974!, G00113360!, G00113361!, LE!—isolectotypes).

### 5.5. Frullania asiatica (S. Hatt.) Mamontov & J. J. Atwood, comb. et stat. nov.

**Basionym**: *Frullania dilatata* subsp. *asiatica* S. Hatt., J. Jap. Bot. 57: 258 (1982).

**Type citation**: India. West Bengal: near Sandakphu, Darjeeling area, 11,600–11,900 ft, on branches of shrub, 26 April, *Z. Iwatsuki, A. J. & Evelyn Sharp B-745/a* (NICH).

**Type specimen**: INDIA: West Bengal, Near Sandakphu, Darjeeling area, 11,900–11,600 ft. alt., 26 April 1965, *Z. Iwatsuki, A. J. & E. Sharp B745a* (NICH-262371!, holotype).

**Illustrations**: Figure 8. Hattori & Thaithong [64] (p. 178, Figure 1).

**Other studied specimen**: INDIA: West Bengal, Darjeeling area, Sikkim border, along Rangeet River on Teesta Road, 9000 ft. alt, 20 April 1965, *Z. Iwatsuki* et al. *8831a* (MHA, MO).

## 6. Conclusions

A characteristic feature of *F.* sect. *Trachycolea* is the presence of caducous leaves; most species of this section (in the present sense) have this feature. The *F. parvistipula* complex is an artificial group, which includes unrelated species of three sections of *F.* subg. *Trachycolea*. All of these species share caducous leaves and *dilatata*-type vegetative morphology; due to this, they often are very difficult to distinguish from one another.

*Frullania* sect. Trachycolea includes *F. appalachiana*, *F. azorica*, *F. caucasica*, *F. conistipula*, *F. dilatata*, *F. eboracensis*, *F. koponenii*, and *F. virginica*, of which five species occur in Eurasia and four in North America, with only one species, *F. caucasica*, in common. In North America, the latter species has been found in the Mountain States (Colorado and New Mexico), while *F. appalachiana*, *F. eboracensis*, and *F. virginica* are endemics of eastern North America. The latter three species are closely related to each other and form a group, in which *F. caucasica* is nested in a sister relationship. In Eurasia, *F. caucasica* is distributed in Southern and Central Europe (Italy and Switzerland), the Caucasus, the Ural Mts., the Western Siberian plains, and the mountains of Southern Siberia (Altai and Sayan mountain systems and the Khamar-Daban Range). This species, however, has still not been found in Eastern Siberia east of the 105th meridian or in East Asia. The sister relationship between *F. caucasica* and the group of eastern North American species may follow from ancient Atlantic migrations of its common ancestor. However, the modern occurrence of *F. caucasica* in North America may be a result of relatively recent, perhaps Pleistocene, migration of this species from Asia through the Beringia land bridge. The possibility of such migrations follows from the close phylogenetic affinity between North American populations of *F. caucasica* and the Siberian ones, rather than the European, as well as the absence of other representatives of the section in western North America. The same way of migration from Asia to North America possibly occurred in the case of *F. parvistipula*, according to the modern occurrence of this species in Arizona, in the proximity to North American localities of *F. caucasica*. Both cases are particular to the phenomenon of the existence of species that are common to Eurasia and North America (both western and eastern).

In Eastern Siberia, the Russian Far East, and Japan, there are two species with *F.* sect. *Trachycolea*, *F. conistipula* and *F. koponenii*, of which *F. koponenii* is related to both *F. caucasica* and the group of North American species. *Frullania koponenii*, therefore, might be an East Asian vicariate of an ancient European species that produced, in time, *F. caucasica* and the *F. appalachiana–eboracensis–virginica* complex. The resurrected *F. conistipula* is phylogenetically most related to *F. dilatata* and probably represents an East Asian vicariate of the latter species. *Frullania dilatata* is widely distributed in Europe and the Caucasus and has isolated localities in Asia Minor; however, both *F. conistipula* and *F. dilatata* have recently been found in the Altai Mts. (Southern Siberia, Russia), at a great distance from the main areas of their modern distribution. Therefore, a complicated history of the distribution of both species and their new findings in Southern Siberia and/or Central Asia might be supposed. The last species of the section, *F. azorica*, is nested in a basal position to all other species of the section and represents an endemic of the Macaronesian–Mediterranean region.

In general, the distribution and phylogenetic relationships of the discussed species, as well as the absence of other representatives of *F.* sect. *Trachycolea* in Southeast Asia, Australasia, Africa, and Central and South America, assume that the crown group of the section originated in Europe or in the Ancient Mediterranean region, possibly due to climatic changes in the Oligocene or Miocene. Further migrations of species of the section into Asia and North America, interrupted by periods of glaciation, could have given rise to the secondary speciation in eastern North America and the north of East Asia.

## Figures and Tables

**Table 1:** List of taxa, specimens’ vouchers, and GenBank accession numbers for tested specimens. Accessions obtained in this study are in bold.

Taxon	Specimen Voucher	GenBank Accession Number	
		ITS1-2 nrDNA	*trn*L-F cpDNA
*F. aberrans*	Russia: Primorye Terr. 1, Mamontov 808-1-4726 (MHA)	**MW358434**	**MW351720**
*F. aberrans*	Russia: Primorye Terr. 2, Mamontov 689-1-4724 (MHA)	no data	**MW351721**
*F. aberrans*	Russia: Primorye Terr, 3, Mamontov 697-1-2 (MHA)	**MW358435/MW358436**	**MW351722**
*F. aberrans*	Russia: Primorye Terr. 4, Mamontov 686-1-4723 (MHA)	**MW358437**	**MW351723**
*F. acutiloba*	India, Schaefer-Verwimp & Verwimp 28265 (GOET)	FJ380421	FJ380257
*F. amplicrania*	Japan, Ohnishi 5840 (HIRO)	FJ380447	FJ380285
*F. anomala*	New Zealand, Engel & von Konrat 27,319 (GOET)	FJ380457	FJ380297
*F. appalachiana*	USA: North Carolina 1, Davison 7888 (UNA)	HQ330382	HQ330418
*F. appalachiana*	USA: North Carolina 2, Davison & Smith 6389 (UNA)	HQ330383	HQ330419
*F. azorica*	Portugal: Madeira 1, Eckstein 487 (GOET)	HQ330386	HQ330422
*F. azorica*	Portugal: Madeira 2, Mues 3743 (SAAR)	HQ330387	HQ330423
*F. azorica*	Portugal: Madeira 3, Schaefer-Verwimp & Verwimp 25,896 (GOET)	FJ380436	FJ380272
*F. baladina*	Fiji: Pocs & Pocs 03259/N (GOET)	FJ380454	FJ380293
*F. brittoniae*	USA: Davison & Kauffman 5334 (GOET)	FJ380442	FJ380278
*F. caucasica* 1	Georgia: Zündorf 21852 (JE), as *F.parvistipula* in Bombosch et al. [14]	HQ330415	no data
*F. caucasica* 1	Russia: Altai Rep., Mamontov 333-1-39 (MHA)	**MW358439**	**MW351725**
*F. caucasica* 1	Russia: Buryatia Rep. 2, Mamontov 639-1-1 (MHA)	**MW358441**	**MW351727**
*F. caucasica* 1	Russia: Krasnodar Terr. 1, Konstantinova K235b-18 (KPABG)	**MW358442**	**MW351728**
*F. caucasica* 1	Russia: Krasnodar Terr. 2, Konstantinova K270-18 (KPABG)	**MW358443**	**MW351729**
*F. caucasica* 1	Russia: Krasnodar Terr. 3, Konstantinova K284-1-18 (KPABG)	**MW358444**	**MW351730**
*F. caucasica* 1	Russia: Krasnodar Terr. 4, Konstantinova K287-18 (KPABG)	**MW358445**	**MW351731**
*F. caucasica* 1	Russia: North Ossetia Rep. 1, Rumyanzeva AR-3-2-11 (KPABG)	**MW358446**	**MW351732**
*F. caucasica* 1	Russia: North Ossetia Rep. 2, Rumyanzeva AR-2-11 (KPABG)	no data	**MW351733**
*F. caucasica* 1	Russia: Tyva Rep., Otnyukova (KPABG-101204)	**MW358448**	**MW351735**
*F. caucasica* 1	USA: Colorado 1, Weber et al. B-112022 (MO)	**MW358450**	**MW351737**
*F. caucasica* 1	USA: Colorado 2, Wittmann et al. B-112078 (MO)	**MW358451**	**MW351738**
*F. caucasica* 1	USA: New Mexico 1, Worthington 31081 (MO, GOET), as *F. parvistipula* in Bombosch et al. [14]	**MW358452/MW358453** HQ330379/FJ380435	**MW351739** FJ380271
*F. caucasica* 1	USA: New Mexico 2, Worthington 32,814 (GOET), as *F. parvistipula* in Bombosch et al. [14]	FJ380433	FJ380269
*F. caucasica* 1	USA: New Mexico 3, Schofield 96,656 (MHA)	**PP239508**	**PP261943**
*F. caucasica* 2	Russia: Perm Terr., Bezgodov AB204a-18 (KPABG)	**MW358447**	**MW351734**
*F. caucasica* 2	Switzerland: Rueegsegger 1303-III (KPABG)	**MW358449**	**MW351736**
*F. caucasica* 2	Russia: Buryatia Rep. 1, Mamontov 560-6-1, 4610 (MHA)	**MW358440**	**MW351726**
*F. caucasica* 3	Italy: Sicily, Eckstein 4684 (GOET), as *F. parvistipula* in Bombosch et al. [14]	FJ380438	FJ380274
*F. conistipula*	Russia: Buryatia Rep., Mamontov 450-1-5592 (MHA)	**MW358454**	**MW351740**
*F. conistipula*	Russia: Primorye Terr. 1, Mamontov 697-1-4584 (MHA)	**MW358455**	**MW351741**
*F. conistipula*	Russia: Primorye Terr. 2, Mamontov 807-1-4522 (MHA)	**MW358456/MW358457**	**MW351742**
*F. conistipula*	Russia: Primorye Terr. 3, Mamontov 807-1-4 (MHA)	**MW358458**	**MW351743**
*F. conistipula*	Russia: Primorye Terr. 4, Mamontov 801-1-9 (MHA)	**MW358459**	**MW351744**
*F. conistipula*	Russia: Sakhalin Reg., Kunashir, Bakalin K-37-5-06 (KPABG-110245)	**MW358460**	**MW351745**
*F. conistipula*	Russia: Trans-Baikal Terr. 1, Afonina A5510-1 (KPABG-116339)	**MW358461**	**MW351746**
*F. conistipula*	Russia: Trans-Baikal Terr. 2, Afonina A5510-8 (KPABG-118647)	**MW358462**	**MW351747**
*F. conistipula*	Russia: Trans-Baikal Terr. 3, Afonina A5710-1 (KPABG-116341)	**MW358463**	**MW351748**
*F. conistipula*	Russia: Trans-Baikal Terr. 4, Mamontov 544-1-6073 (MHA)	**PQ213807**	**PQ213858**
*F. davurica*	Russia: Primorye Terr., Gambaryan VLA-h-764 (GOET)	FJ380444	FJ380280
*F. dilatata*	Bulgaria: Hentschel 0758 (GOET)	FJ380434	FJ380270
*F. dilatata*	Georgia: Bordshomi, Zündorf 21940 (JE)	HQ330390	HQ330425
*F. dilatata*	Germany: Saarland, Sesterhenn 5474 (SAAR)	HQ330396	HQ330430
*F. dilatata*	Germany: Nordrhein-Westfalen, Mamontov 244-12-1 (MHA)	**MW358465**	**MW351750**
*F. dilatata*	Italy: Apulia, Sauer 3090 (GOET)	HQ330401	HQ330435
*F. dilatata*	Russia: Adygeia Rep., Konstantinova K472-1-07 (KPABG-111725)	**PP239515**	**PP261950**
*F. dilatata*	Russia: Altai Terr. 1, Mamontov 219-15 (KPABG)	**MW358466**	**MW351751**
*F. dilatata*	Russia: Altai Terr. 2, Mamontov 219-18 (KPABG)	**MW358467**	**MW351752**
*F. dilatata*	Russia: Altai Terr. 3, Mamontov 219-32-8 (KPABG, MHA)	**MW358468**	**MW351753**
*F. dilatata*	Spain: Konstantinova K12-9a-19 (KPABG)	**MW358470**	**MW351755**
*F. dorsimamillosa*	China: Koponen 45098 (JE), as *F. fuscovirens* var. *gemmipara* in Hentschel et al. [10]	FJ380440	FJ380276
*F. duthieana*	Bhutan: Miehe & Miehe 00-132-21 (GOET), as *F. duthiana* in Hentschel et al. [10]	FJ380443	FJ380279
*F. eboracensis*	Canada: Prince Edward Island, Belland	HQ330409	HQ330442
*F. eboracensis*	USA: Alabama, Davison 6875 (UNA)	HQ330410	HQ330443
*F. eboracensis*	USA: New York, Smith 50725 (UBC)	HQ330414	HQ330446
*F. eboracensis*	USA: Davison 5193 (GOET)	FJ380437	FJ380273
*F. ericoides*	Virgin Islands, Gradstein 6421 (GOET)	FJ380405	FJ380240
*F. fugax*	New Zealand: Engel & von Konrat 24698 (GOET)	FJ380455	FJ380295
*F. fukuzawana*	Russia: Primorye Terr. 1, Mamontov 851-1-6016 (MHA, KPABG-126113)	**PP239511**	**PP261946**
*F. fukuzawana*	Russia: Primorye Terr. 2, Mamontov 850-1-6021 (MHA, KPABG-126089)	**PP239512**	**PP261947**
*F. fukuzawana*	Russia: Primorye Terr. 3, Mamontov 850-1-6301 (MHA, KPABG-126112)	**PP239513**	**PP261948**
*F. fukuzawana*	Russia: Primorye Terr. 4, Mamontov 855-1-6303 (MHA, KPABG-126088)	**PP239514**	**PP261949**
*F. fukuzawana*	Russia: Sakhalin Reg., Kunashir, Mamontov 815-1-5628 (MHA, KPABG-126092)	**PP239510**	**PP261945**
*F. ignatovii*	Russia: Buryatia Rep., Mamontov 384-8 (KPABG-120318, isotype)	MT408599	no data
*F. jackii*	Switzerland: Urmi & Schaefer-Verwimp 16,328 (GOET)	FJ380445	FJ380281
*F. koponenii*	Russia: Buryatia Rep. 1, Mamontov 565-3-4730 (MHA)	**MW358471**	**MW351756**
*F. koponenii*	Russia: Buryatia Rep. 2, Mamontov 658-1-1 (MHA)	**MW358472**	**MW351757**
*F. koponenii*	Russia: Primorye Terr. 1, Mamontov 692-1-1 (MHA)	**MW358473**	**MW351758**
*F. koponenii*	Russia: Primorye Terr. 2, Mamontov 805-1-1 (MHA)	**MW358474**	**MW351759**
*F. koponenii*	Russia: Sakhalin, Bakalin S-24-18-06 (KPABG)	**MW358475**	**MW351760**
*F. koponenii*	Russia: Sakhalin Reg., Kunashir, Mamontov 837-1-6005 (MHA, KPABG-126091)	**PP239509**	**PP261944**
*F. koponenii*	Russia: Trans-Baikal Terr. 1, Mamontov 61-1 (LE)	**MW358476**	no data
*F. koponenii*	Russia: Trans-Baikal Terr. 2, Mamontov 511-1-2 (MHA)	**MW358477**	**MW351761**
*F. koponenii*	Russia: Trans-Baikal Terr. 3, Afonina A07208 (KPABG-113885)	no data	**MW351762**
*F. moniliata*	Japan: Ohnishi, 5346 (HIRO)	FJ380500	FJ380346
*F. ‘muscicola’*	Russia: Primorye Terr. 1, Mamontov 739-3-1 (MHA)	**MW358478**	**MW351763**
*F. ‘muscicola’*	Russia: Primorye Terr. 2, Mamontov 805-1-4 (MHA)	**MW358479**	**MW351764**
*F. ‘muscicola’*	Russia: Trans-Baikal Terr., Mamontov 264-6 (MHA)	**MW358480**	no data
*F. parvistipula*	China: Yunnan Prov., 1, Mamontov 722-1-4 (MHA)	**MW358485**	**MW351768**
*F. parvistipula*	China: Yunnan Prov., 2, Mamontov 737-1-2 (MHA)	**MW358486**	**MW351769**
*F. parvistipula*	Russia: Buryatia Rep., Mamontov 376-1-1 (KPABG)	**MW358487**	**MW351770**
*F. parvistipula*	Russia: Trans-Baikal Terr., Mamontov 182-6-1 (KPABG-118618)	**MW358488**	**MW351771**
*F. parvistipula*	USA: Arizona 1, Brinda 9978 (MO)	**MW358489**	**MW351772**
*F. parvistipula*	USA: Arizona 2, Brinda 1398 (MO)	**MW358490**	**MW351773**
*F. parvistipula*	USA: Arizona 3, Brinda 1396b (MO)	**MW358491**	**MW351774**
*F. parvistipula*	China: Guangxi 1, Mamontov 716-1-1 (MHA)	**MW358492**	**MW351775**
*F. parvistipula*	China: Guangxi 2, Mamontov 719-1-1 (MHA)	**MW358493**	**MW351776**
*F. pedicellata*	Japan: Inoue s.n., as ‘No. 705. *Frullania muscicola* Steph.’ of Bryophyta Selecta Exsiccata (KPABG)	**MW358481/MW358482**	**MW351765**
*F. plana*	USA: Davison 4325 (GOET)	FJ380431	FJ380267
*F. reptans*	New Zealand: Engel & von Konrat 24,974 (GOET)	FJ380450	FJ380288
*F. riparia*	USA: Worthington 29,965 (GOET)	FJ380446	FJ380284
*F. subcaduca*	Papua New Guinea, Norris 62427 (JE)	no data	FJ380283
*F. taradakensis*	Russia: Primorye Terr. 1, Mamontov 801-1-5 (MHA)	**MW358483**	**MW351766**
*F. taradakensis*	Russia: PrimoryeTerr. 2, Bakalin P-69-10-06 (KPABG-110319)	**MW358484**	**MW351767**
*F. virginica*	USA: Louisiana, Hyatt s.n. (GOET)	HQ330416	HQ330447
*F. virginica*	USA: Alabama, Davison 3550 (GOET)	HQ330417	HQ330448
*F. dilatata* × *caucasica*	Russia: Chechen Rep., Doroshina 467-2-18 (KPABG)	**MW358469**	**MW351754**

**Table 2:** Values of infrageneric and infraspecific *p*-distances of ITS2/*trn*L-F for the selected species of *F.* subg. *Trachycolea* (%), n/c—non calculated value due to single specimen only.

	Taxon	Infraspecific p-Distances, ITS2/*trn*L-F (%)	Infrageneric p-Distances, ITS2/*trn*L-F (%)																
			1	2	3	4	5	6	7	8	9	10	11	12	13	14	15	16	17
1	*caucasica* 1	0.4/0.2																	
2	*caucasica* 2	0.1/0.1	2.1/0.8																
3	*caucasica* 3	n/c/n/c	1.9/1.2	1.2/1.4															
4	*virginica*	0.2/0.0	4.0/1.5	4.1/1.8	3.0/1.5														
5	*appalachiana*	0.4/0.2	3.7/1.3	3.9/1.5	2.1/1.5	2.5/0.7													
6	*eboracensis*	0.2/0.0	3.6/0.9	3.9/1.0	2.9/1.3	2.3/0.6	1.8/0.5												
7	*koponenii*	1.2/0.0	3.7/1.1	3.6/1.5	2.5/1.2	3.6/1.6	3.2/1.5	3.5/1.4											
8	*conistipula*	0.3/0.0	4.4/0.9	4.7/1.3	2.5/1.0	4.9/1.4	4.2/1.3	4.5/1.2	3.4/1.1										
9	*dilatata*	0.4/0.3	4.0/0.6	3.9/0.9	3.1/0.8	4.1/1.1	3.4/1.0	3.9/0.9	3.0/0.7	3.0/0.5									
10	*azorica*	0.2/0.0	7.0/1.4	6.2/1.6	6.2/1.5	6.8/1.9	6.3/1.9	6.8/1.7	6.2/1.7	6.6/1.5	5.4/1.1								
11	*parvistipula*	0.8/0.3	11.2/2.5	10.8/2.5	9.6/2.4	10.6/3.1	10.6/3.2	10.3/2.7	10.6/2.7	11.7/2.6	10.0/2.2	11.5/3.0							
12	*baladina*	n/c/n/c	10.0/2.9	7.5/3.2	9.9/2.6	9.8/2.9	9.9/3.3	9.9/3.1	10.0/3.0	10.4/2.8	9.8/2.5	11.8/3.2	3.4/0.9						
13	*reptans*	n/c/n/c	10.7/2.7	8.0/3.0	10.6/2.4	10.4/3.1	10.6/3.1	10.6/2.9	10.8/2.8	11.2/2.6	10.1/2.3	12.6/3.1	3.4/0.7	3.8/0.9					
14	*fugax*	n/c/n/c	8.9/2.7	7.1/3.0	8.8/2.1	8.7/3.1	8.8/3.1	8.8/2.9	8.9/2.8	9.2/2.7	8.3/2.3	9.9/3.1	5.1/1.2	6.7/1.5	6.0/1.3				
15	*anomala*	n/c/n/c	10.0/3.0	7.9/3.4	10.3/2.8	10.0/3.5	9.2/3.5	9.7/3.2	10.4/3.2	10.6/3.0	9.8/2.7	9.9/3.1	7.8/1.4	8.2/1.7	8.6/1.5	7.3/1.7			
16	*fukuzawana*	0.1/0.0	16.4/3.1	15.3/3.4	13.1/3.1	16.4/3.7	16.3/3.7	15.4/3.5	15.6/3.3	16.9/3.1	15.8/2.7	16.3/3.5	16.5/2.9	13.8/2.9	14.0/3.1	12.9/2.7	15.0/3.5		
17	*‘muscicola’*	0.4/0.0	15.0/3.1	13.9/3.6	12.2/2.7	15.0/3.6	15.2/3.7	14.7/3.4	14.4/3.4	15.8/3.2	14.5/2.9	14.4/3.8	14.2/3.1	13.0/3.1	12.5/3.3	12.1/2.9	14.3/3.3	9.1/2.7	
18	*aberrans*	0.5/0.0	17.4/2.4	15.0/2.8	16.1/2.0	16.9/2.9	17.5/2.9	17.4/2.7	17.0/2.6	17.9/2.5	17.5/2.1	18.5/3.1	15.9/2.2	15.8/2.2	16.3/2.4	15.3/2.4	16.8/2.8	13.6/1.9	12.2/2.0

## Data Availability

Data supporting reported results can be partially found in the Tropicos database (https://www.tropicos.org/home) and the publicly archived dataset of “L” Information system (https://isling.org/, accessed on 8 July 2024).
